# The Transcriptional Repressor TupA in *Aspergillus niger* Is Involved in Controlling Gene Expression Related to Cell Wall Biosynthesis, Development, and Nitrogen Source Availability

**DOI:** 10.1371/journal.pone.0078102

**Published:** 2013-10-29

**Authors:** Doreen Schachtschabel, Mark Arentshorst, Benjamin M. Nitsche, Sam Morris, Kristian F. Nielsen, Cees A. M. J. J. van den Hondel, Frans M. Klis, Arthur F. J. Ram

**Affiliations:** 1 Institute of Biology Leiden, Leiden University, Molecular Microbiology and Biotechnology, Leiden, The Netherlands; 2 Applied and Molecular Microbiology, Institute of Biotechnology, Berlin University of Technology, Berlin, German; 3 Department for Systems Biology, Technical University of Denmark, Lyngby, Denmark; 4 Swammerdam Institute for Life Sciences, Amsterdam of University, Amsterdam, The Netherlands; 5 Kluyver Centre for Genomics of Industrial Fermentation, Delft, The Netherlands; Seoul National University, Republic of Korea

## Abstract

The Tup1-Cyc8 (Ssn6) complex is a well characterized and conserved general transcriptional repressor complex in eukaryotic cells. Here, we report the identification of the Tup1 (TupA) homolog in the filamentous fungus *Aspergillus niger* in a genetic screen for mutants with a constitutive expression of the *agsA* gene. The *agsA* gene encodes a putative alpha-glucan synthase, which is induced in response to cell wall stress in *A. niger.* Apart from the constitutive expression of *agsA*, the selected mutant was also found to produce an unknown pigment at high temperatures. Complementation analysis with a genomic library showed that the *tupA* gene could complement the phenotypes of the mutant. Screening of a collection of 240 mutants with constitutive expression of *agsA* identified sixteen additional pigment-secreting mutants, which were all mutated in the *tupA* gene. The phenotypes of the *tupA* mutants were very similar to the phenotypes of a *tupA* deletion strain. Further analysis of the *tupA-17* mutant and the *ΔtupA* mutant revealed that TupA is also required for normal growth and morphogenesis. The production of the pigment at 37°C is nitrogen source-dependent and repressed by ammonium. Genome-wide expression analysis of the *tupA* mutant during exponential growth revealed derepression of a large group of diverse genes, including genes related to development and cell wall biosynthesis, and also protease-encoding genes that are normally repressed by ammonium. Comparison of the transcriptome of up-regulated genes in the *tupA* mutant showed limited overlap with the transcriptome of caspofungin-induced cell wall stress-related genes, suggesting that TupA is not a general suppressor of cell wall stress-induced genes. We propose that TupA is an important repressor of genes related to development and nitrogen metabolism.

## Introduction

The fungal wall is an essential organel. It forms a strong structural barrier that offers protection against mechanical damage, helps to withstand the internal turgor pressure, and maintains and determines the shape of the cell. Developmental stages or dimorphic switches strongly affect the composition of the cell wall, both in structure as well as in the type of cell wall mannoproteins that are incorporated into the cell wall [1]–[4]. The cell wall also contributes to invasion of sturdy substrates, and the formation of multi-cellular structures. The structural components of the wall mainly consist of polysaccharides, such as polymers of glucose (β-1,3- and β-1,6-glucan, and chitin, which consists of β-1,4-linked N-acetyl-glucosamine residues [5], [6]. In addition, filamentous fungal walls including those of *Aspergillus* species often contain α-glucans, β-1,3-1,4-glucan, galactomannan, galactosaminogalactan and galactomannoproteins [7]–[9]. The actual cell wall composition not only depends on the fungal species, but its composition is also highly dependent on environmental factors and developmental stages [10].

Many (pathogenic) fungi are able to switch from yeast to filamentous growth. This is accompanied by major changes in cell wall composition. The dimorphic switch has been extensively studied in *Candida albicans* and this has shown that the expression of cell wall genes is highly dynamic during the yeast to hyphal transition [11], [12]. Moreover, in pathogenic dimorphic fungi like *Histoplasma capsulatum, Cryptococcus neoformans, Blastomyces dermatitidis*, and *Paracoccidioides brasiliensis*, the virulent yeast form contains substantial levels of α-glucan (35–46% of total cell wall carbohydrates) in comparison to dramatically decreased levels in the avirulent mycelial form [13]–[17]. In addition, cell wall stress conditions have been reported to induce important changes in cell wall composition by the specific induction of cell wall remodeling genes or cell wall proteins [18]–[20]. The Cell Wall Integrity (CWI) pathway, a conserved signaling pathway, is one of the most important pathways mediating this response (see [21]–[23] for reviews). In *A. niger*, activation of the CWI pathway results in a strong transcriptional induction of *agsA*, an alpha-glucan synthase-encoding gene [20], [24]. The induced transcription of *agsA* in response to cell wall stress is mediated via a highly conserved Rlm1p-like MADS-box transcription factor protein, called RlmA [24].

The Tup1-Cyc8(Ssn6) complex is a general transcriptional co-repressor complex that controls the expression of genes involved in various processes. This complex is especially well studied in the yeast *Saccharomyces cerevisiae*, and mutational and genome-wide expression studies have shown that Tup1 is responsible for the repression of over 180 genes, including gene sets regulated by glucose, DNA damage, mating type, and oxygen availability, and gene sets involved in osmotic stress responses, flocculation, and dimorphism [25]–[28]. Recent studies have shown that the repressor function of Tup1-Cyc8 is caused by the interaction of the complex with a specific DNA-binding domain, thereby preventing the recruitment of transcriptional co-activators [29], [30]. The important role of Tup1/TupA in pathogenic fungi has received further attention because of its important role in dimorphism and pathogenicity. Although the role of Tup1 in fungal dimorphism is conserved, the way it controls the switch differs between fungi [12], [31]–[33].

We previously reported about the isolation of UV-mutants showing a constitutive high expression of the *agsA* gene by selection for improved growth on acetamide as sole nitrogen source and for the presence of GFP-labeled, fluorescent nuclei [34]. For this, a dual reporter strain was used that contained a construct with the *amdS* sequence (coding for an acetamidase) and the Histone2B-GFP sequence both cloned behind an *agsA* promoter region. In this study, we describe a mutant with a constitutive expression of the *agsA* gene and show that the mutant is mutated in the *A. niger* TupA homolog. The *tupA* (An15g00140) mutant in *A. niger* displays in addition to induced expression of *agsA* a strongly reduced radial growth rate, increased branching, and abundant secretion of an unknown pigment into the medium. We present further genome-wide transcriptomic consequences of the mutation in the co-repressor complex and focus on the impact of *tupA* on the transcriptional control of cell wall biosynthetic genes in *Aspergillus niger*. The genome-wide study combined with phenotypic analysis of the *tupA* strains also suggests that TupA is an important repressor of genes related to nitrogen metabolism, which might explain the important role of TupA in relation to dimorphic switching in dimorphic fungi.

## Materials and Methods

### Strains, Plasmids, Cosmids, and Growth Conditions

The *A. niger* strains used in this study are listed in Table 1. Strains were grown on minimal medium (MM) [35] containing 1% (w v^−1^) glucose or on complete medium (CM), containing 0.5% (w v^−1^) yeast extract and 0.1% (w v^−1^) casamino acids in addition to MM-glucose. When required, plates or medium were supplemented with 10 mM uridine, SDS (50 µg/ml), Calcofluor White (50–400 µg/ml), caspofungin (0.2–1.5 µg/ml), or with sorbitol (1.2 M) to assay growth. MM agar plates containing acetamide as sole nitrogen source were made as described [36].

**Table 1:** Strains used in this study.

Strain	Description	Reference
N402	*cspA1* derivative of ATCC9029	[89]
AB4.1	*pyrG* ^−^ derivative of N402	[90]
MA169.4	*kusA::DR-amdS-DR pyrG* ^−^	[43]
RD15.8	pPagsA-H2B-GFP-TtrpC-pyrG* and p*PagsA-amdS-TamdS*/pAN7.1	[34]
RD15.8#36	pPagsA-H2B-GFP-TtrpC-pyrG* and p*PagsA-amdS-TamdS*/pAN7.1	[34]
DSC12	*pyrG* ^−^ derivative of RD15.8#36	this study
DSC13	DSC12 containing pMA172-An15g00140 (*tupA*)	this study
MA245.1	*ΔtupA*::*pyrG* in MA169.4	this study
MA246.1	*ΔtupA*::*pyrG* in RD15.8 *pyrG* ^−^	this study
SM2.36	*ΔtupA*::*pyrG* AB4.1 *pyrG* ^−^	this study

Targeted integration of constructs at the *pyrG* locus using the pyrG* allele was done as described [37]. *E. coli* DH5α strains were transformed by electroporation for propagation and amplification of the cosmids. Amplification of plasmid DNA was performed using the XL1-Blue strain, which was transformed using the heat-shock protocol as described by [38]. Transformation of *A. niger* was performed as described by Meyer *et al*. [39] using 40 mg lysing enzyme (L-1412, Sigma, St. Louis) per gram wet weight of mycelium. *A. niger* genomic DNA (including cosmid DNA) was isolated as described previously [39]. [α-^32^P]dCTP-labeled probes were synthesized using the Rediprime II DNA labeling system (Amersham Pharmacia Biotech, Piscataway, NJ) according to the instructions of the manufacturer. All molecular techniques were carried out as described [40]. Sequencing was performed by Macrogen Europe (Amsterdam, The Netherlands).

### Bioreactor Cultivations

Bioreactor cultivations were carried out as described previously. Fermentation medium (FM) adjusted to pH 3, is composed of 0.75% glucose, 0.45% NH_4_Cl, 0.15% KH_2_PO4, 0.05% KCl, 0.05% MgSO_4_, 0.1% trace element solution and 0.003% yeast extract as described [41]. Freshly harvested conidia (5×10^9^) from strain N402 and RD15.8#36 were used to inoculate 5 liters of FM. Cultivations were performed in a BioFlo3000 bioreactor (New Brunswick Scientific), where the temperature, pH (set to 3), and agitation speed were controlled online using the program NBS Biocommand. The cultivation program consisted of two consecutive phases: (i) 30°C, agitation speed of 250 rpm, and headspace aeration, for the first 5 h; (ii) 30°C, agitation speed of 750 rpm, and sparger aeration during the second phase. Mycelial samples were taken after specific time points for microarray, metabolic, and microscopic analyses.

### Identification and Cloning of *tupA*

RD15.8#36 was made *pyrG* ^−^ by selecting 5-fluoroorotic acid (5-FOA)-resistant mutants as described [39]. One of the generated *pyrG* ^−^ mutants was transformed with a genomic cosmid library in an AMA1-containing *pyrG*-based self-replicating vector [40]. Cosmids from transformants that show complementation of the growth-deficient phenotype at 30°C were isolated. To re-isolate the complementing cosmid, DNA from transformants was isolated and used for transformation to *E. coli* DH5α by electroporation. Fresh ampicillin (amp)-resistant transformants were transferred to 50 ml LB-amp medium and grown overnight at 37°C, and cosmid DNA was isolated using a small-scale isolation protocol essentially as described (Sambrook *et al.,* 1989). Primers cosT7 and cosUL were used for sequencing the borders of the insert [34]. Subclones were generated by digestion of the cosmids with various enzymes, and fragments were ligated into properly digested pBluescriptSK(+). The generated subclones were co-transformed with pAB4.1 to RD15.8#36 *pyrG* ^−^. The 10.1-kb *Hind*III subclone giving complementation was subject to further analysis and sequencing.

For complementation studies the An15g00140 locus, including approximately 2.3-kb promoter and 1.4-kb terminator regions, was PCR-amplified using N402 genomic DNA as template and the *Not*I site-containing primers P1_0140_For and P2_0140_Rev (Table 2). The fragments were cloned into pJET1.2 (Fermentas), sequenced, released from pJET1.2 via *Not*I restriction and cloned into *Not*I-linearized pMA172 [43]. Respective plasmids (pMA172-0140 and pJET-0140) were then transformed into RD15.8#36 *pyrG* ^−^. Primary transformants containing the complementation plasmid were isolated on MM without uridine supplementation and further analyzed by Southern blot. To allow pMA172 plasmid loss, spores were streaked on MM containing 10 mM uridine. The DNA sequence of the An15g00140 gene in the parental strain (RD15.8), in the wild-type strain (N402) and in the mutant strains was determined by sequencing. The An15g00140 locus with a 0.7-kb promoter and a 0.7-kb terminator region was amplified with primers P3_0140_For and P8_0140_Rev (Table 2), using genomic DNA of the respective three strains as template DNA. PCR products were directly sequenced with appropriate primers (Table 2).

**Table 2:** Overview of the primers used in this study.

Primer	Sequence 5′-3′	used for	remark
P1_0140_For	aaggaaaaaagcggccgcTGAAGTGCCAGCCAGTAGTGG	amplifying locus	*Not*I underlined
P2_0140_Rev	aaggaaaaaagcggccgcTGGGTGATCGTGACTTTACCGCGGTAAAGTCACGATCACCCA	amplifying locus and generating deletion construct 3′	*Not*I underlined
P3_0140_intI	CGGTCACACTAAGCGCCGTA	sequencing *tupA* alleles	
P4_0140_intII	CGACACAAATCTTTCGCGCTA	sequencing *tupA* alleles	
P5_0140_intIII	TTGCCTGACCTCTGACCTCG	sequencing *tupA* alleles	
P6_0140_intIV	GCTGGCAATGGTCGGTACA	sequencing *tupA* alleles	
P7_0140_intV	CGGAAATGCGCAGATGATG	sequencing *tupA* alleles	
P8_0140_intVI	CGAGAGATTGCATGGCAGC	sequencing *tupA* alleles	
P9_0140_ For	cccaagcttACATGATTTGCTGGCTCCGAC	generating deletion construct	*Hind*III underlined
P10_0140_ Rev	ccgctcgagAGCTGAGGCTGAAGGAGGAG	generating deletion construct	*Xho*I underlined
P11_0140_ For	ggggtaccTGAAGTGCCAGCCAGTAGTGG	generating deletion construct	*Kpn*I underlined

### Generation of a *tupA* Deletion Strain

An An15g00140 gene disruption cassette, *(*Δ*tupA::pyrG)*, was prepared by a three-way ligation. 5′ and 3′ regions flanking the coding region were amplified by PCR using the primers listed in Table 2. Fragments were cloned into pJet2.1. The 0.5-kb 5′ region *Kpn*I/*Xho*I fragment, the 0.5-kb 3′ *Hind*III/*Not*I fragment and a 1.7-kb *Hind*III/*Xho*I fragment from pAO4-13 [44] containing the *A. oryzae pyrG* gene, were cloned into the pBluescript-SK+ backbone prepared by digestion with *Kpn*I and *Not*I to give Δ*tupA::pyrG*.

Using Δ*tupA::pyrG* as a template, a PCR with primers P1_0140_For and P11_0140_Rev was performed and the linear deletion fragment was transformed to MA169.4 *pyrG* ^−^ (ku70::DR-amdS-DR *pyrG* ^−^). Uridine prototrophic transformants were purified twice on MM and subjected to Southern blot analysis [39]. DNA extraction was carried out from mycelia that had been collected from liquid MM cultures containing 0.003% yeast extract.

### Microarray and Northern Analysis

#### (i) RNA isolation and quality control

Culture broth samples (10 ml each) obtained from the above-described bioreactor cultures were quickly harvested and filtered, and the mycelial samples were immediately frozen using liquid nitrogen. Total RNA for Northern and microarray analyses was isolated from frozen, ground mycelium by Trizol extraction according to the manufacturer’s instructions. Following extraction, RNA was purified on NucleoSpin RNA II columns (Machery-Nagel), including a DNase I digestion step. RNA was eluted in 60 µl of MilliQ water. RNA quantity and quality were determined on a Nanodrop spectrophotometer, and integrity was tested on an Agilent 2100 Bioanalyser. The spectrum generated by the Agilent Bioanalyser was visually inspected for possible RNA degradation and contamination with genomic DNA to ensure good sample quality.

### Microarray Analysis and Bioinformatics

Microarray analyses for N402 and RD15.8#36 were performed on mycelia obtained during the exponential growth phase from two independent bioreactor cultivations (biological duplicates), when 75% of glucose had been consumed. Probe synthesis and fragmentation were performed at ServiceXS (Leiden, Netherlands) according to the GeneChip Expression Analysis Technical Manual (Affymetrix Inc. 2002). DSM (Delft, Netherlands) proprietary *A. niger* gene chips were hybridized, washed, stained, and scanned as described in the Gene-Chip Expression Analysis Technical Manual (Affymetrix Inc. 2002). The generated transcriptomic data set and description of the Affymetrix gene chip used are deposited at the Gene Expression Omnibus database and can be accessed via their accession numbers GSE50523 and GPL6785, respectively. For transcriptomic data analysis, the statistical programming language R as used including open source and open development packages of the Bioconductor project [45]. Affymetrix probe-level data from CEL files were preprocessed using the Robust Multi-Array average (RMA) [46] algorithm as implemented in the Affy package [47]. For transcripts targeted by multiple probe sets, average expression values were computed prior to the identification of differentially expressed genes with the limma package [48]. The proportion of false positives was controlled by calculating the false discovery rate (FDR) according to the method of Benjamini and Hochberg [49] and controlled at 0.005 without a minimal fold change criterion. Applying a critical FDR of 0.05, Gene Ontology (GO) [50] enrichment analysis for differentially expressed gene sets was performed using Fisher’s exact Test Gene Ontology annotation tool (FetGOat) [51] including its most recent GO annotation.

## Results

### Phenotypic Analysis of *A. niger* Mutant RD15.8#36

**Figure 1:**
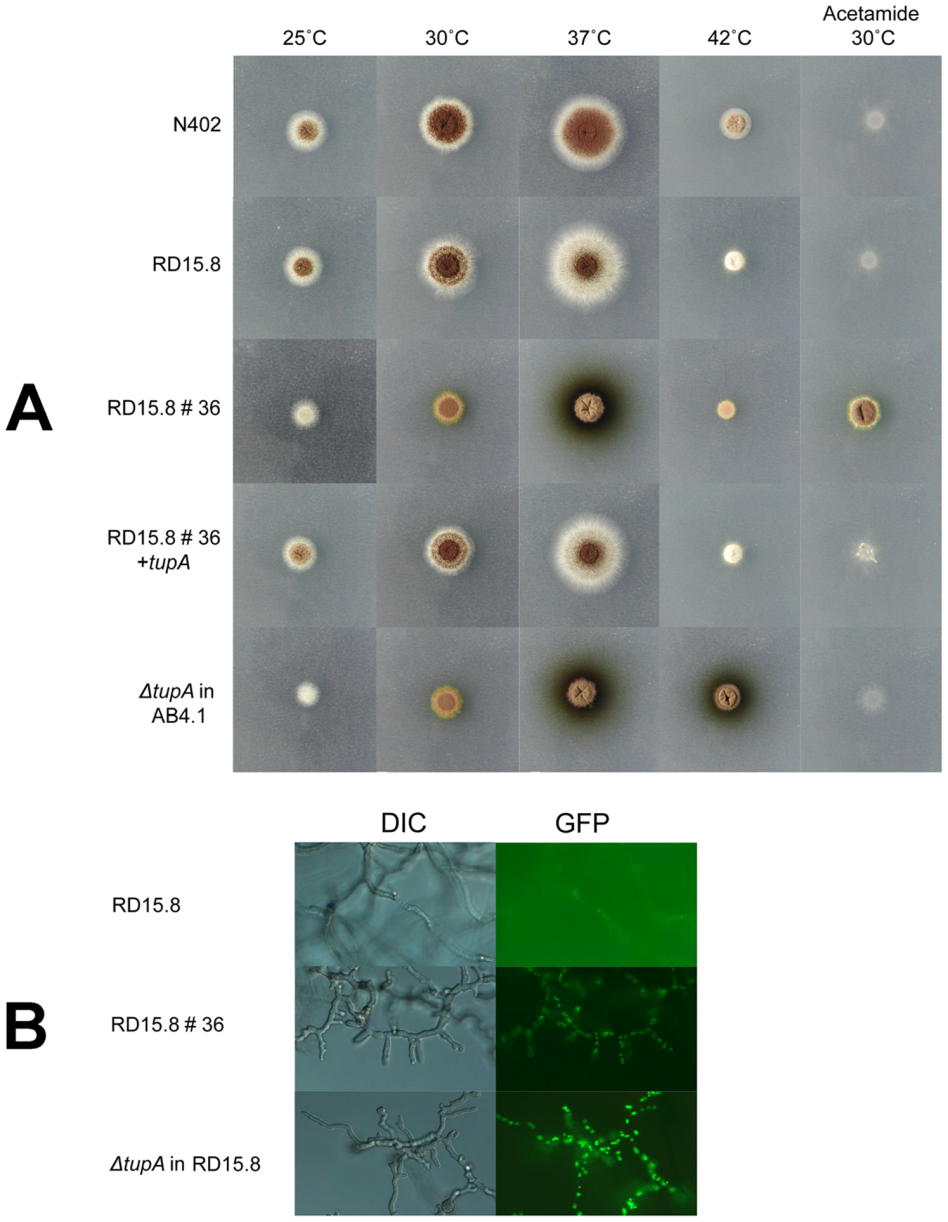
Phenotypic characterization of the *tupA* mutants. (a) Ten thousand spores of UV mutant RD15.8#36, the parental strain RD15.8, the complemented mutant RD15.8#36/pAn14g00140, the full deletion mutant Δ*tupA* and wild-type N402, were spotted and growth was monitored on MM-glucose-nitrate plates or MM/glucose-acetamide plates acetamide (first vertical column or MM-glucose plates (if not stated differently) at the indicated temperature for 3 days. (b) DIC- and fluorescent pictures of parental strain (RD15.8), the *tupA* mutant, and the *tupA* deletion strain in the RD15.8 background after growth for 20 hours at 30°C in MM with casamino acids.

We previously reported the use of a genetic screen to isolate mutants with an induced expression of *agsA*, a gene encoding a putative α-glucan synthase. In this screen, a reporter strain is used that contains two reporter constructs: the *agsA* promoter fused to the *A. nidulans* acetamidase (*amdS*) gene and the *agsA* promoter fused to H2B-GFP [34]. Since *agsA* is specifically induced in response to cell wall stress conditions [20], this screen is expected to yield cell wall mutants with a constitutively activated cell wall integrity pathway resulting in *agsA* expression. The *agsA-amdS* reporter allows direct selection for mutants that can grow on acetamide and the second reporter was included to check for cis-acting mutations in the *agsA-amdS* promoter region and for mutants in which the expression of endogenous acetamidase was deregulated. In comparison to the parental strain (RD15.8), mutant RD15.8#36 shows clear induction of the two *agsA* reporter constructs, which results in strongly improved growth on acetamide medium at 30°C and nuclei that show enhanced green fluorescence, consistent with increased expression of the *agsA* gene (Figure 1a and 1b). The mutants showed several growth-related phenotypes including retarded spore germination (germination of RD15.8#36 occurred about 12 h later than in the parental strain (data not shown), and a strongly reduced radial growth rate (Figure 1a). Interestingly, at 37°C, the mutant secreted an enhanced amount of dark green- to brown-colored pigment in the medium (Figure 1a). We did not observe increased sensitivity of the mutant towards the cell wall-perturbing compounds caspofungin and Calcofluor White (CFW) or to the cell membrane-perturbing agents SDS and fenpropimorph, an inhibitor of ergosterol biosynthesis (data not shown), which would have been indicative of defects in cell wall synthesis [52], [53]. Attempts to improve the growth of the RD15.8#36 mutant by supplementing the culture medium with 1.2 M sorbitol were only partly successful (data not shown). This suggests that despite the induced expression of *agsA* the mechanical strength of the mutant wall was not significantly affected.

### The *A. niger* Homolog of the General Transcriptional Repressor TupA Complements *A. niger* Mutant RD15.8#36

To identify the mutated gene causing the phenotypes of the RD15.8#36 mutant, a *pyrG* ^−^ derivative was made and transformed with a *pyrG*-based genomic cosmid library [42]. Initially, circa 1,000 uridine-prototrophic transformants were directly selected for enhanced growth at 30°C and one hundred of those were purified and retested for wild-type growth at 30°C on acetamide and minimal medium. Cosmids were isolated from ten transformants with a strong complementation phenotype and *EcoR*I restriction of the cosmids revealed the isolation of identical cosmid clones that could complement the RD15.8#36 phenotypes. Sequence analysis of the insert borders revealed the presence of two different chromosomal fragments, which complicated identification of the responsible gene. Hence, the isolated cosmid was restricted with various enzymes and individual fragments were co-transformed with pAB4.1 to RD15.8#36*pyrG* ^−^. A 10.1-kb *Hind*III fragment that complemented the reduced growth rate was subsequently cloned and sequenced. The *Hind*III subclone harbored a single full-length predicted open reading frame, namely, An15g00140. This gene was amplified via PCR and ligated into the autonomously replicating vector pMA172 [43] and the resulting plasmid was transformed to RD15.8#36*pyrG* ^−^. As shown in Figure 1a, An15g00140 complemented the different phenotypes of the RD15.8#36. This indicates that a single gene is responsible for these phenotypes.

Comparison of the protein sequence generated from the An15g00140 gene revealed that the protein displays strong sequence similarity to the transcriptional repressor RcoA of *A. nidulans* and to Tup1 of *S. cerevisiae*, and we will refer to this gene as *tupA*.

### Characterization of *tupA* Mutants in the Cell Wall Mutant Collection

**Figure 2:**
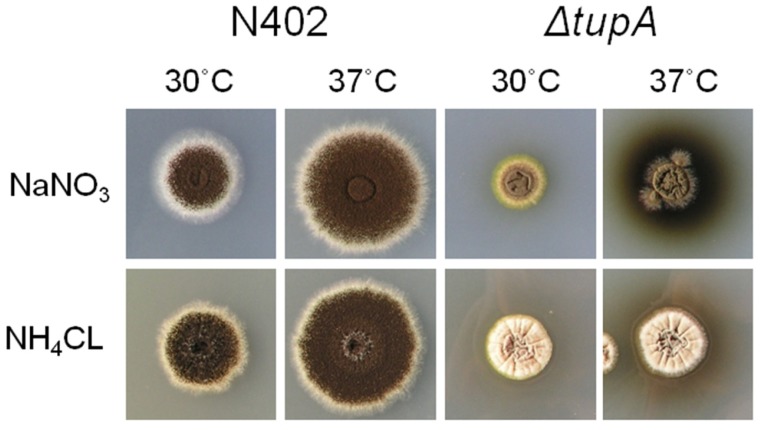
The effects of growth temperature and nitrogen source on pigment formation in the *tupA* mutant. High temperatures (37°C) and nitrate are required for optimal production of the pigment. Note also the strong reduction of conidiospores on top of the vegetative mycelium in the *tupA* mutant grown on ammonium as nitrogen source. Pictures taken after 3 days.

The 240 cell wall mutants of *A. niger* that were isolated via a genetic screen for high *agsA* expression [34] and yielded the identification of TupA as described above, was screened for pigment-producing mutants at high temperature. Apart from mutant RD5.18#36, sixteen more mutants were identified that secreted the pigment at high temperature in a nitrogen source-dependent manner (data not shown). To verify whether these 17 mutants were all mutated in *tupA* or otherwise affected in *agsA* expression, the *tupA* locus of each of the mutant was PCR amplified and sequenced. All mutants were mutated in the *tupA* gene (Table 3). We were unable to PCR amplify the *tupA* locus from mutant *tupA-14* indicating rearrangement of the *tupA* locus in this strain. A variety of mutations including point mutations, insertions, deletions, missense mutations and mutations affecting intron splicing were detected. Tup1 is known to interact with Cyc8/Ssn6 and deletion of either *tupA* or *cys8/ssn6* leads to similar phenotypes [26]. Because of the saturating high number of *tupA* mutants identified by screening and the lack of any other mutant with a similar phenotype but not *tupA*, we suspect that mutations in the Cyc8/Ssn6 homolog in *A. niger* (An02g03940) do not lead to a strong induction of the *agsA* promoter. The sequencing of the mutant alleles also confirms that in our original mutants the *tupA* locus was mutated and that *tupA* was not acting as a suppressor gene in our complementation studies. To investigate the consequences of a loss of function of the *tupA* gene in *A. niger*, a gene deletion vector (*p*Δ*tupA::pyrG*) was constructed, in which the ORF was replaced by the *A. oryzae pyrG* gene. After transformation, primary transformants were purified twice on MM lacking uridine, and stable transformants were obtained. Proper disruption of the *tupA* gene in several transformants was confirmed by Southern blot analysis (data not shown). The phenotypes of the Δ*tupA* deletion mutant were similar to *tupA* mutant (RD15.8#36) (Figure 1a and b). The phenotype of the *tupA* deletion strain was characterized on glucose medium containing either nitrate or ammonium as a nitrogen source. The phenotype was characterized by a reduced radial growth rate (approximately 50% reduced after 7 days of incubation) and a strong reduction (∼80%) in conidiation in the presence of ammonium but not in the presence of nitrate (Table 4). We also noticed that the production of the pigment was highest when the *tupA* mutant grew at high temperatures (37°C) on nitrate. Equivalent amounts on ammonium (10 mM) strongly repressed pigment formation (Figure 2).

**Table 3:** Overview of mutations in *A niger tupA* mutants.

Strain	Allel	Position of mutation inORF (including introns)	Consequence of the mutation (s)		Protein sequence(WT: 1–590)
RD6.47#28	*tupA-1*	GA deletion (61, 62)	out of frame after aa 21	truncation	1–21
RD6.13#22	*tupA-2*	TA to AT (1561, 1562)	ATT to ATA (silent I remains I);AAG to TAG (K to stop)	truncation	1–358
RD6.13#53	*tupA-3*	A to C (487)	mutation in 3′ intron splice siteTAG to TCG	truncation	1–60
RD15.4#2	*tupA-4*	G to C (2351)	TGG to TCG (W to S)	point mutation	1–590 (W to S at 575)
RD15.4#3	*tupA-5*	G to A (889)	mutation in 3′ intron splice siteCAG to CAA	truncation	1–170
RD15.4#24	*tupA-6*	extra T (1355)	Out of frame after aa 316	truncation	1–316
RD15.4#26	*tupA-7*	extra T (786)	Out of frame after aa 160	truncation	1–160
RD15.4#27	*tupA-8*	C to T (2128)	TCT to TTT (S to F)	point mutation	1–590 (S to F at 489)
RD15.4#29	*tupA-9*	A to T (1772)	AAG to TAG (K to stop)	truncation	1–473
RD15.4#37	*tupA-10*	C to T and C deletion(669, 670)	GGC to GGT (Silent G remains G);out of frame after aa 122	truncation	1–122
RD15.4#47	*tupA-11*	TG to GA (1664, 1665)	TGG to GAT (W to D)	point mutation	1–590 (W to D at 393)
RD15.4#49	*tupA-12*	C to T (43)	CAA to TAA (Q to stop)	truncation	1–14
RD15.4#52	*tupA-13*	T to C (2350)	TTG to CCG (W to R)	point mutation	1–590 W to R at 575)
RD15.4#60	*tupA-14*	no PCR product obtained			
RD15.8#13	*tupA-15*	C to T (1856)	TCG to TTG (S to L)	point mutation	1–590 (S to L at 457)
RD15.8#27	*tupA-16*	G to A (2125)	GGT to GAT (G to D)	point mutation	1–590 (G to D at 488)
RD15.8#36	*tupA-17*	G to A (2144)	TGG to TGA (W to stop)	truncation	1–519

**Table 4:** Growth and sporulation analysis of wild-type (N402) and *ΔtupA* (*tupA::pyrG* in MA169.4) strains after 7 days of growth.

Strain	N-source	Temp.(°C)	Colonydiameter (cm)	Spores percolony	Spores/cm^2^ *	Relativegrowth	Relativesporulation
WT	ammonium	30	10.0	2.0×10^8^	2.5×10^6^	100%	100%
WT	ammonium	37	14.0	6.8×10^8^	4.4×10^6^	100%	100%
WT	nitrate	30	9.8	2.0×10^8^	2.6×10^6^	100%	100%
WT	nitrate	37	14.9	3.0×10^8^	1.7×10^6^	100%	100%
*ΔtupA*	ammonium	30	5.8	0.16×10^8^	0.61×10^6^	58.0%	24.4%
*ΔtupA*	ammonium	37	7.3	0.33×10^8^	0.79×10^6^	52.1%	18.0%
*ΔtupA*	nitrate	30	5.2	0.68×10^8^	3.2×10^6^	53.1%	123%
*ΔtupA*	nitrate	37	5.5	0.49×10^8^	2.1×10^6^	36.9%	124%

* Number of spores of the colony/((Radius of the colony)^2^×π).

### Microarray Analysis of Bioreactor-grown Wild-type and Mutant Strains

**Figure 3:**
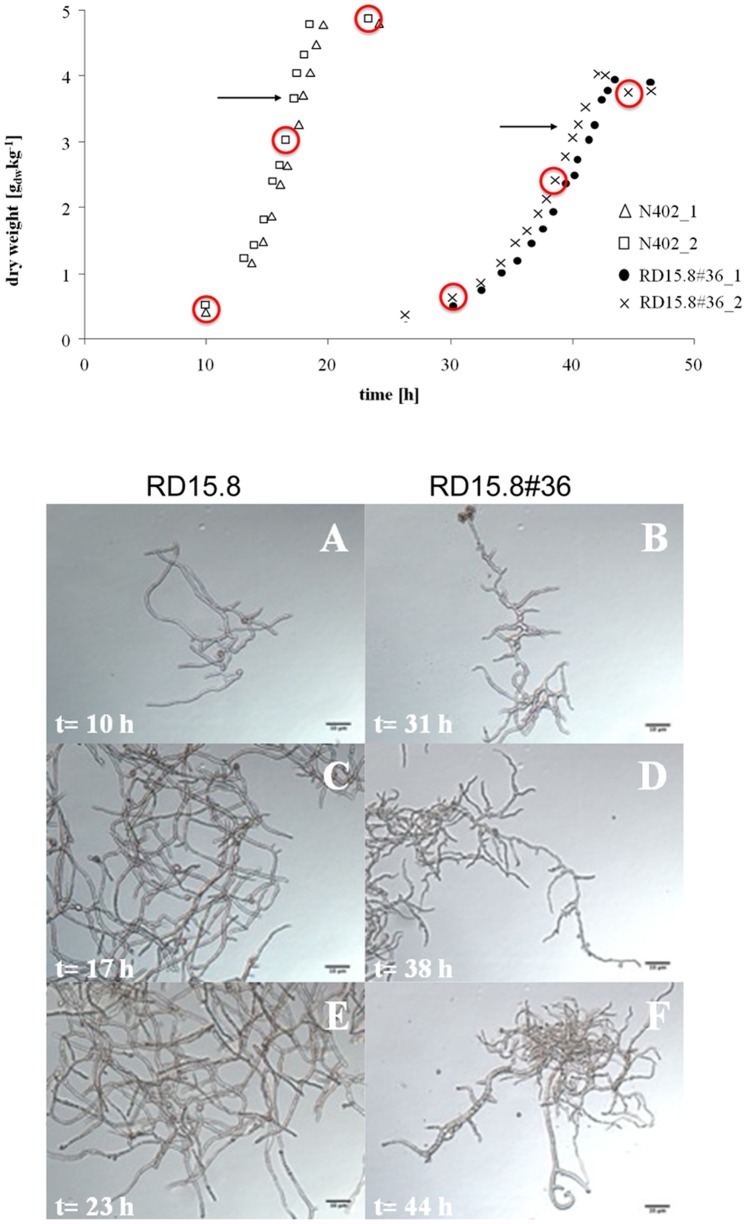
Physiological and morphological of *A. niger* RD15.8#36 in comparison to wild-type N402. (Top figure) Biomass accumulation in the duplicate cultures and the maximum specific growth rate (µ_max_). The arrows indicate the time point when 75% of glucose was consumed and mycelia were harvested for transcriptomic analysis. (Lower figure (A-F) Morphology of the mutant RD15.8#36 in bioreactor cultures in comparison to the wild-type N402 both grown on FM. Mycelium from the exponentially growing culture was harvested at the indicated time points. Scale bar, 10 µm.

To identify genes in *A. niger* that are differentially expressed in the *tupA* mutant compared to the wild-type strain, RNA samples were extracted from the wild-type (N402) and the *tupA* mutant strain (RD15.8#36). To ensure controlled and reproducible growth conditions, both N402 and RD15.8#36 were cultivated in a bioreactor (see Materials and Methods). As shown in Figure 3, initial spore germination of the *tupA* mutant was delayed compared to the wild-type strain. The dry weight measurements were used to determine the maximal specific growth rate of both strains under the given growth conditions and this showed that the growth rate of the *tupA* mutant strain (0.16 h^−1^) was substantially lower than that of the wild-type strain N402 (0.25 h^−1^) (data not shown), consistent with the substantial decrease in the radial growth rate when cultured on a solid surface (Figure 1 and 2). The final growth yields (4.0 g_DW_ kg^−1^ for the mutant and 4.8 g_DW_ kg^−1^ for N402) differed only moderately and were highly reproducible. At the end of the exponential growth phase the supernatant of the mutant culture turned dark-green to brown whereas the wild-type broth remained colorless. The green-brownish color at this time point was not due to the presence of melanized conidiospores as microscopic observation did not indicate the presence of spores. Conceivably, the metabolic expenditure required for the formation and secretion of these secondary metabolites by the mutant strain might explain its lower biomass yield. As shown in Figure 3 (panels A-F), also under these growth conditions the *tupA* mutant showed morphological aberrations, which included increased branching, increased variability in hyphal diameters, and curled hyphae. In addition, RD15.8#36 sporadically formed swollen, abnormally shaped, branched hyphae (Figure 3. These morphologic differences might also result in less efficient metabolism and therefore reduced biomass yield.

RNA was isolated from both cultures when 75% of the carbon source (glucose) was converted into biomass (3.2 g_DW_ kg^−1^ for RD15.8#36, 3.7 g_DW_ kg^−1^ for N402). Two biological replicates for each strain were performed corresponding to four microarray analyses in total.

### Genome-wide Expression Analysis of the *tupA* Mutant in Comparison to the Wild-type Strain

To identify the processes in *A. niger* affected by the *tupA* mutation, we first confirmed that the biological reproducibility of gene transcription for the biological duplicates was high, as evidenced by a mean relative standard deviation of 0.05 for both the wild-type and mutant, respectively, and similar to values reported previously [54], [55]. Two thousand and eight out of 14,165 *A. niger* genes (using a low corrected P-value of <0.005) were differentially expressed (as defined in Materials and Methods). The large size of this set of genes (∼14%) corroborates the importance of *tupA* as a general transcriptional repressor. Of the 2008 differentially expressed genes 1053 were higher expressed in the *tupA* mutant compared to wild-type (tupA^UP^) and 955 genes were down-regulated (tupA^DOWN^). A comprehensive list of all differentially expressed genes including statistical significance and transcript ratios is presented in.

For more insight into the processes that are affected in the *tupA* mutant, we performed GO-enrichment analysis of the genes that were significantly higher expressed in the *tupA* mutant using FetGOat [51]. The GO-terms (Biological Process) overrepresented in the *tupA* mutant include 28 GO terms. The GO-terms are given in Table S2. Among those 28 BP terms, 9 terminal nodes were present which are summarized in Table 5. The presence of GO term ´related to carbon metabolic procesś (acetate and pentose metabolic processes), ´related to differentiation and development´ (conidium formation, reproductive processes and sporulation), ´related to stress responsé (oxidative stress) suggest conservation of TupA function among fungi in repressing gene sets under non-inducing conditions.

**Table 5:** GO terms (Biological Process) enriched among up-regulated genes in the *tupA* strain.

	Description		FDR
GO:0006083	acetate metabolic process	BP	7,13E-04
GO:0044282	small molecule catabolic process	BP	7,46E-03
GO:0015695	organic cation transport	BP	1,95E-02
GO:0075307	positive regulation of conidium formation	BP	2,65E-02
GO:2000243	positive regulation of reproductive process	BP	3,03E-02
GO:0019321	pentose metabolic process	BP	3,36E-02
GO:0006979	response to oxidative stress	BP	3,95E-02
GO:0045881	positive regulation of sporulation resulting in formation of a cellular spore	BP	3,95E-02
GO:0033609	oxalate metabolic process	BP	4,29E-02

About half of the differentially expressed genes were down-regulated in the *tupA* mutant and also for these genes a GO enrichment analysis was performed. Sixty-seven GO terms (BP-terms) were enriched, which are summarized in Table S3. Among the sixty-seven enriched GO-terms, 19 terminal nodes were present, which are summarized in Table 6. Most of the terms are associated with metabolic and biosynthetic process, which may be caused by the slower growth rate of the *tupA* mutant. The inactivation of TupA not only affects the expression of a large number of genes, but the fold-changes of many of these genes were also remarkable. Seventy-five genes were higher expressed in the *tupA* strain with a FC>10 and 77 genes were down-regulated with a FC>10 (Tables S4 and S5).

**Table 6:** GO terms (Biological Process) enriched among down-regulated genes in the *tupA* strain.

	Description		FDR
GO:0009108	coenzyme biosynthetic process	BP	3,56E-03
GO:0046355	mannan catabolic process	BP	4,92E-03
GO:0006534	cysteine metabolic process	BP	8,41E-03
GO:0006879	cellular iron ion homeostasis	BP	9,53E-03
GO:0019184	nonribosomal peptide biosynthetic process	BP	1,10E-02
GO:0042364	water-soluble vitamin biosynthetic process	BP	1,64E-02
GO:0006544	glycine metabolic process	BP	1,99E-02
GO:0006066	alcohol metabolic process	BP	2,16E-02
GO:0071577	zinc ion transmembrane transport	BP	2,92E-02
GO:0006733	oxidoreduction coenzyme metabolic process	BP	2,92E-02
GO:0043156	chromatin remodeling in response to cationstress	BP	2,92E-02
GO:0019662	non-glycolytic fermentation	BP	2,92E-02
GO:0009082	branched chain family amino acidbiosynthetic process	BP	3,19E-02
GO:0000301	retrograde transport, vesicle recyclingwithin Golgi	BP	3,36E-02
GO:0006555	methionine metabolic process	BP	4,12E-02
GO:0015812	gamma-aminobutyric acid transport	BP	4,12E-02
GO:0008219	cell death	BP	4,12E-02
GO:0010106	cellular response to iron ion starvation	BP	4,45E-02
GO:0015893	drug transport	BP	4,92E-02

In the following sections we will focus on genes related to synthesis of the fungal cell wall and to development and highlight some of the most significant changes in gene expression (Tables 7–10).

**Table 7:** Cell wall biosynthetic genes up-regulated in the *tupA* mutant.

Gene number	Gene name	Description	*tupA*	WT	FC	P-value
An08g03580	*bgtA*	Putative beta-1,3-glucanosyltransferase (GH17-family)	1669	37	44.6	2.28E-07
An18g04100	*exgA*	Putative exo-beta-1,3-glucanase (GH5-family)	907	45	20.1	1.46E-06
An02g03980	*kslA*	Putative transglycosidase required for beta-1,6 glucan biosynthesis; ScKre6-like	179	32	5.5	3.21E-06
An01g06500	*dfgD*	Putative endo-mannanase with a possible role in GPI-CWP incorporation; ScDfg5-like(GH76-family)	152	29	5.2	1.02E-06
An16g02850	*crhF*	Putative transglycosidase involved in cell wall biosynthesis (GH16-family)	673	135	5.0	5.78E-06
An02g09050	*gelG*	GPI- anchored beta-1,3-glucanosyltransferase	362	78	4.6	2.21E-05
An05g00130	*knlA*	Putative transglycosidase required for beta-1,6 glucan biosynthesis; ScKre9-like	1008	219	4.6	1.31E-06
An15g07800	*agtC*	GPI-anchored alpha-glucanotransferase	85	21	4.1	9.14E-06
An07g04650	*bgtB*	Putative beta-1,3-glucanosyltransferase (GH17-family)	381	120	3.2	5.76E-06
An15g07810	*agsB*	Putative catalytic subunit alpha-glucan synthase complex	82	31	2.7	5.40E-04
An01g12450	*bxgA*	Putative exo-beta-1,3-glucanase (GH55-family)	1122	473	2.4	5.87E-05
An08g09030	*cfcB*	Putative ClassV Chitinase (GH18)	57	24	2.4	1.01E-04
An07g07530	*crhB*	Putative transglycosidase involved in cell wall biosynthesis (GH16-family)	1589	753	2.1	9.72E-04
An10g00400	*gelA*	GPI- anchored beta-1,3-glucanosyltransferase	2918	1435	2.0	1.02E-04
An09g04010	*chsB*	Putative chitin synthase Class III	1574	819	1.9	4.51E-04
An02g10490	*–*	Putative endo-1,3(4)-beta-glucanase	457	262	1.7	5.18E-04
An03g05260	*csnA*	similarity to chitosanase csnA - *Aspergillus oryzae*	72	44	1.6	9.15E-04
An01g04560	*mlgA*	strong similarity to mixed-linked glucanase precursor MLG1 - *Cochliobolus carbonum*	194	125	1.6	4.33E-03
An02g13180	*bgxB*	Putative exo-beta-1,3-glucanase (GH55-family)	38	25	1.5	1.56E-03
An07g01540	*rotA*	Protein with putative role in beta-1,6 glucan biosynthesis	735	506	1.5	2.39E-03

**Table 8:** Predicted (GPI-anchored) cell wall protein encoding genes up-regulated in the *tupA* mutant.

Genenumber	Genename	Description	PredictedGPI-anchor	*tupA*	WT	FC	P-value
An15g07790		putative cell wall protein; serine/threonine rich	yes	2412	32	74.4	4.40E-07
An11g02730		putative cell wall protein; serine/threonine rich	yes	743	23	32.5	1.68E-07
An02g09010		putative cell wall protein; serine/threonine rich	yes	1464	48	30.3	2.75E-07
An14g02100	*cwpA*	hydrogen fluoride extractable GPI-anchored cell wall protein	yes	1433	97	14.7	1.75E-06
An16g07920		putative cell wall protein	yes	5261	403	13.1	9.09E-07
An16g01780		putative cell wall protein	yes	9574	808	11.8	3.41E-06
An06g01000		putative cell wall protein; serine/threonine rich	yes	4951	699	7.1	1.47E-05
An12g00140		putative cell wall protein; serine/threonine rich	yes	763	113	6.8	9.53E-07
An18g06360		putative cell wall protein; serine/threonine rich	yes	689	137	5.0	9.96E-07
An07g04620		putative cell wall protein	yes	2155	493	4.4	3.28E-04
An01g05230		putative cell wall protein; serine/threonine rich	yes	6536	1940	3.4	7.03E-06
An07g06210		putative cell wall protein; serine/threonine rich	yes	738	231	3.2	4.29E-05
An14g01820	*phiA*	strong similarity to cell wall protein binB - *Aspergillus nidulans*	no	1966	611	3.2	1.02E-04
An02g00120		putative cell wall protein serine/threonine rich	yes	771	403	1.9	1.69E-03
An18g03730		putative cell wall protein serine/threonine rich	yes	3086	1895	1.6	1.24E-03

**Table 9:** Cell wall biosynthetic genes down-regulated in the *tupA* mutant.

Genenumber	Genename	Description	*tupA*	WT	FC-down	P-value
An11g01240	*dfgH*	Putative endo-mannanase (GH76-family) with a possible role in GPI-CWP incorporation	42	503	12.0	1.67E-07
An09g06400	*ctcA*	Predicted GPI-anchored protein. Putative ClassIII Chitinase (GH18)	161	1852	11.5	3.26E-07
An08g09610	*agnD*	Putative alpha-1,3-glucanase GH71	324	2187	6.7	8.15E-07
An06g00360	*dfgF*	Putative endo-mannanase (GH76-family) with a possible role in GPI-CWP incorporation	131	753	5.8	8.84E-07
An07g01160	*crhC*	Predicted GPI-anchored protein. Putative transglycosidase of GH16-family	153	584	3.8	8.69E-06
An08g07350	*gelB*	Predicted GPI-anchored protein. Putative 1,3-beta-glucanosyltransferase GH72	224	713	3.2	1.78E-05
An09g00670	*gelD*	Predicted GPI-anchored protein. Putative 1,3-beta-glucanosyltransferase GH72	1424	4169	2.9	8.65E-06
An04g04670	*cfcC*	Putative ClassV Chitinase (GH18)	232	609	2.6	3.08E-05
An13g02510	*crhE*	Predicted GPI-anchored protein. Putative transglycosidase of GH16-family	83	213	2.6	4.88E-05
An01g11010	*crhD*	Predicted GPI-anchored protein. Putative transglycosidase of GH16-family	256	641	2.5	6.91E-05
An12g10380	*chsE*	Putative chitin synthase ClassIII	1203	2556	2.1	3.43E-04
An16g08090	*dfgE*	Putative endo-mannanase (GH76-family) with a possible role in GPI-CWP incorporation	281	597	2.1	1.44E-04
An12g02450	*agsC*	Putative catalytic subunit alpha-glucan synthase complex	161	323	2.0	1.63E-04
An08g05290	*chsG*	Putative chitin synthase ClassVI	28	55	2.0	8.78E-05
An02g07020	*cfcA*	Putative ClassV Chitinase (GH18)	132	258	2.0	5.38E-04
An11g07660	*exgB*	Putative exo-1,3-beta-glucanase	144	272	1.9	2.36E-04
An09g03070	*agsE*	Putative catalytic subunit alpha-glucan synthase complex	376	699	1.9	1.88E-03
An09g06260	*agnC*	Putative alpha-1.3-glucanase GH71	355	571	1.6	9.68E-04
An12g02460	*agtB*	GPI-anchored alpha-glucanotransferase	328	483	1.5	2.48E-03
		Cell wall signaling				
An04g10140	*mltB*	Putative plasma membrane sensor required for cell wall integrity signalling	47	235	5.0	3.27E-05

**Table 10:** Predicted GPI-anchored cell wall protein-encoding genes down-regulated in the *tupA* mutant.

Gene number	Gene name	Description	*tupA*	WT	FC	P-value
An07g05670		Putative cell wall protein	196	342	1.7	6.45E-04
An12g07750		Putative cell wall protein serine threonine rich	141	1076	7.6	1.89E-03
An16g07950		Putative cell wall protein serine threonine rich	109	7139	65.4	6.98E-08

### Differential Expression of Cell Wall Biosynthesis and Remodeling Genes

The *A. niger tupA* mutant was identified in a plate screen for mutants with an increased expression of *agsA*. We therefore looked specifically if the *agsA* gene was also induced in the microarray data set. Surprisingly, the *agsA* gene was not induced in the *tupA* mutant under the growth conditions used in the bioreactor (normalized expression of *agsA* in the *tupA* mutant: 294 vs normalized expression wild-type: 254; FC 1.2; FDR 2.45E-01). Indeed, Northern analysis of additional samples taken from the bioreactor runs confirmed that *agsA* was not induced during the bioreactor cultivation (data not shown), whereas the expression of *agsA* on plate was clearly induced. We investigated whether nitrogen source was influencing the expression of *agsA* as the plate screen was carried out on minimal medium containing acetamide as a nitrogen source whereas the bioreactor cultivation was carried out in minimal medium containing ammonium as a carbon source. To also examine the effect of the pH, spores of the *tupA* mutant containing the *PagsA*-H2B-GFP reporter were allowed to germinate in nitrate (pH3.0 and pH6.0) and ammonium (pH3.0 and pH6.0) medium and the fluorescence was analyzed. Under all condition, we observed fluorescent nuclei to a similar strength, indicating that neither the low pH nor the nitrogen source explained for the absence of *agsA* expression in the bioreactor (Figure S1). It is important to note that the bioreactor condition represents only a single condition and thus only a small fraction of the genes affected by the *tupA* deletion will be revealed. For example transcriptomic consequences in relation to the loss of TupA such as conidiation, carbon stress, nitrogen stress, effects of pH and DNA damage conditions will not be detected. Further experiments are required to understand in detail the effects of loss of TupA repression in general and of *agsA* expression in particular. Several factors, including the physiology of the fungus (exponential growth in the bioreactor and a mix of exponential growing mycelium (edge) and stationary phase mycelium (central parts of the colony) [56], [57], or different expression dependent on the water content [58]–[59] could account for these differences. The morphological differences of the *tupA* strain still raised the question whether the transcription of other cell wall formation-associated genes was altered in the *tupA* strain. To examine this, the cell wall-related genes that were annotated as such [60] were examined. This analysis identified 20 up-regulated genes involved in the biosynthesis of cell wall polysaccharides (Table 7). Especially, genes related to β-glucan processing were significantly up-regulated, but several genes involved in chitin and α-glucan synthesis were up-regulated in the *tupA* mutant as well (Table 7). Two genes (*bgt1*/An08g03580 and *exgA*/An18g04100), both enzymes involved in β-glucan synthesis, were very highly expressed in the *tupA* mutant. In addition, the expression of genes predicted to encode structural (GPI-anchored) cell wall proteins [60] were analyzed (Table 8). Fifteen cell wall protein-encoding genes were higher expressed indicating that the cell wall protein composition has changed in the *tupA* mutant. One of these proteins, CwpA, has been shown to encode a GPI-anchored cell wall protein that is expressed during stationary phase, but before conidiation markers such as *brlA* and *rodA* [61]. The higher expression of *bgt1* and *cwpA* in the *tupA* mutant was also confirmed by Northern blot analysis (Figure S2).

In addition to higher expressed cell wall-related genes, 19 genes encoding cell wall biosynthetic enzymes and three genes encoding GPI-anchored cell wall proteins were lower expressed in the *tupA* mutant (Table 9 and 10 respectively). Most dramatically down-regulated are a putative endo-mannanase of the DFG family, a GPI-anchored chitinase, a putative alpha-glucanase (*mutA*) and a GPI-anchored cell wall protein of unknown function. The differential expression of *mutA* was confirmed by Northern blot analysis (Figure S2). Collectively, the observations presented in Tables 7, 8, 9, 10 strongly indicate that TupA plays an important role in regulating the formation and remodeling of the cell wall.

### Differential Expression of Asexual Development and Secondary Metabolite Production Related Genes

GO enrichment analysis identified the differential expression of asexual development-related genes. In Table 11 the genes and a short description of the gene products are presented. Four transcription factors (*flbC*, *flbD, brlA*, and *proA*) are significantly up-regulated in the *tupA* mutant during exponential growth. The first three are positive transcription factors required for conidiation in *A. nidulans* (see [62], [63] for reviews) and for *brlA* we confirmed that this gene is required for conidiation in *A. niger* [64]. The ProA transcription factor is homologous to the Pro1/NosA transcription factor which is required for sexual development in *Sordaria macrospora* and a repressor of sexual development in *Aspergillus nidulans* [65], [66]. The genes *ppoA* and *ppoC* encode two fatty acid oxygenases that are required for the production of oxylipins called psi-factors. In *A. nidulans*, psi factors have been shown to alter the ratio of asexual to sexual sporulation. The lack of synthesis of psi-factor in *ppo* disruption strains increased and misregulated the activation of sexual development [67], [68] and has been shown to affect *brlA* expression levels.

**Table 11:** Developmental genes up-regulated in the *tupA* mutant.

Genenumber	Genename	Description	TupA*	WT*	FC	P-value
		**Regulation of Development**				
An02g05420	*flbC*	Putative regulator containing two zinc-finger motifs	672	221	3.0	1.04E-04
An01g04830	*flbD*	strong similarity to myb-like DNA binding protein, required for conidiation	1916	83	23.2	3.20E-07
An01g10540	*brlA*	BrlA C2H2 Zn (II) finger transcription factor required for conidiation	158	22	7.1	6.61E-06
An04g07400	*proA*	Zn(II)2Cys6- transcriptional activator similar to Pro1	597	135	4.4	1.68E-04
An05g00480	*stuA*	APSES-transcription factor (spatial expression of abaA)	2202	870	2.5	7.07E-04
An17g01580	*steA*	Transcriptional Activator containing homeodomain DNA binding; STE12-LIKE	427	231	1.8	1.80E-04
An02g09610	*nsdD*	GATA-transcription factor, light regulation	199	115	1.7	4.03E-04
An01g13660	*abr2*	Putative laccase possible role in pigment biosynthesis	891	28	31.7	7.97E-08
An04g05880	*ppoA*	Fatty acid oxygenase for Psi factor production	211	86	2.5	4.18E-04
An02g07930	*ppoC*	Fatty acid oxygenase for Psi factor production	661	90	7.4	1.72E-06
An12g00710	*esdC*	Required for sexual development in *A. nidulans* negative regulation of conidium formation, positive regulation of sexual sporulation resulting in formation of a cellular spore	3989	804	5.0	3.94E-05
An14g01820	*phiA*	Strong similarity to hypothetical cell wall protein binB; caspofungin induced	1966	611	3.2	1.02E-04
An08g05100	*veA*	Velvet activator induces sexual reproduction *A. nidulans*	319	124	2.6	4.15E-05
An12g03660		CAAX-prenyl cysteine carboxymethyltransferase; a-factor modification; STE14-LIKE	272	114	2.4	7.99E-05
An02g03160	*flbA*	Regulator of G-protein signalling	167	83	2.0	3.02E-04
An18g06110	*rgsA*	Regulator of G-protein signalling	180	96	1.9	4.91E-04
An14g02970	*fphA*	Red light phytochrome An14g02970 AN9008.2 89.m01927 20173.m00405	83	46	1.8	3.85E-03
		**Hydrophobins**				
An15g03800	*hypF*	Putative hydrophobin	948	22	42.7	5.52E-08
An07g03340	*hypE*	Putative hydrophobin	1726	136	12.7	2.34E-07
An01g10940	*hypA*	Putative hydrophobin	125	43	2.9	2.62E-05
An04g08500	*rodA*	Hydrophobin: strong similarity to rodletless protein rodA - *Aspergillus nidulans*	185	88	2.1	1.34E-03
		**Melanin biosynthesis**				
An09g05730	*fwnA*	polyketide synthase required for melanin synthesis *A. niger*	54	128	0.4	3.94E-04
An14g05350	*olvA*	strong similarity to hypothetical yellowish-green 1 ayg1 - *Aspergillus fumigatus*	363	85	4.3	3.42E-05
An14g05370	*brnA*	strong similarity to cell surface ferroxidase precursor Fet3 - *Saccharomyces cerevisiae*	52	34	1.5	1.91E-03
		**Growth and Morphology**				
An16g03740	*pkaR*	protein kinase A regulatory subunit	469	286	1.6	1.59E-03
An01g02320	*rasA*	RAS protein, small GTP binding protein	1938	1217	1.6	7.34E-04

Several hydrophobin genes were up-regulated in the *tupA* mutant (Table 11, Figure S2). However, the hydrophobin gene that is induced during conidiation in response to carbon starvation during zero growth conditions (An03g02360) [41] was not induced in the *tupA* mutant. Finally, we noticed that two genes that are required for spore-related melanin production in *A. niger* (*olvA* and *brnA*) were up-regulated. However, the polyketide synthase (*fwnA*), which is required for the melanin production [69], was not higher expressed. As described above, the loss of repression of transcription due to the *tupA* mutation has an important effect on the expression of genes that regulate and coordinate asexual development. However, not all the genes that are induced during asexual development were induced. We therefore suggest that TupA assists in repression of these genes under non-inducing conditions. For other genes, like *fwnA*, a specific activator is probably required to induce expression. It is important to note that we did not observe formation of asexual structures (conidiospores) during the exponential growth phase of the *tupA* mutant when cultivated in the bioreactor, indicating that not the entire asexual developmental program was turned on in the *tupA* mutant during exponential growth. Also noticeable is the up-regulation of two genes related to cAMP signaling (RasA and PkaR), which hints to an increased activation of cAMP synthesis in the *tupA* mutant.

Differentiation is closely linked to the production of secondary metabolites [70] and therefore the expression of genes and gene clusters potentially encoding secondary metabolite synthesis was also analyzed. To do so, the list of 376 secondary metabolite-related genes in *A. niger* as published by Pel and co-workers was used [60]. Fifteen genes were found to be up-regulated (Table S6). One of them does not belong to any known gene cluster (An02g00840) and is predicted to encode a non-ribosomal protein synthase (NRPS). The 14 remaining up-regulated genes belong to predicted gene clusters. In all cases, only a limited number of the genes in an annotated gene cluster were induced. The gene cluster bordered by genes An08g03730 and An08g03820 includes 6 induced genes of the 10 genes in total in the cluster. An08g03770, which belongs to this cluster and is strongly (31-fold) induced, is predicted to encode a Zn(II)2Cys6 transcription factor. It remains to be elucidated which secondary metabolite is produced by this cluster. In total 34 secondary metabolite genes were down-regulated in the *tupA* mutant, belonging to 17 clusters and three down-regulated genes were not clustered. Similarly, as observed for the *tupA*-induced secondary metabolite genes, not all genes from a cluster were down-regulated. All five genes in the cluster bordered by An03g03520 and An03g03560 showed a strong down-regulation in the *tupA* mutant (Table S6).

### TupA is Involved in Controlling Expression of Extracellular Proteases

The genes *pepA* and *pepB*, both encoding extracellular proteases in *A. niger* [42], are highly expressed in the *tupA* mutant during exponential growth on glucose and ammonium, whereas their expression is low in the wild-type strain. Their respective fold-changes are 224 and 99 (Table S7). Expression of *pepA* and *pepB* requires the Zn_2_Cys_6_ transcription factor called PrtT [42]. Thirteen PrtT targets (including *pepA* and *pepB*) have been described in patent application US 2008/0108105 A1. These PrtT-dependent proteases were identified because their expression is dependent on a functional *prtT* gene; in the *prtT* knock out strain, these proteases are significantly lower expressed. Of the thirteen PrtT targets, 6 genes (including *pepA* and *pepB*) were up-regulated in the *tupA* mutant. The expression of the remaining four genes was less dramatically different between the *tupA* strain and the wild-type strain (Table S7).

## Discussion

It is well established that the Tup1/Cyc8 complex functions as an important repressor complex in eukaryotic cells. Transcriptional analysis of a *A. niger tupA* mutant reveals the important role for TupA in controlling gene expression as about 14% of the genes are differentially expressed (up- or down-regulated) in the *tupA* mutant. Some of these differences could be indirectly caused by the slower growth rate of the *tupA* mutant compared to the wild-type, but the detailed analysis of the genes differentially expressed in the *tupA* mutants (Tables 7, 8, 9, 10, 11, and Tables S6 and S7) suggest that TupA is also important in regulating some specific processes related to cell wall biosynthesis, development, secondary metabolism, and nitrogen regulation.

The *tupA* mutant identified in this study was isolated in a cell wall mutant screen. The selection is based on the observation that *agsA* is induced in response to cell wall stress [71]. This screen has been successful in establishing that galactomannan biosynthesis and functional vacuolar ATPase activity are required for cell wall biosynthesis [34], [72]. The mutants identified (*ugmA* and *vmaD*, respectively) showed several cell wall-related phenotypes such as increased sensitivity towards CFW and SDS. The mutant selected for this study showed a relative strong induction of the *agsA* reporters as observed by its ability to grow relatively well on acetamide, and displayed a relatively strong GFP fluorescence signal compared to other mutants (data not shown). However, the mutant did not show increased sensitivity towards cell wall- or cell membrane-perturbing compounds, suggesting that cell wall integrity was not significantly affected.

Caspofungin, an inhibitor of beta-1,3-glucan synthase, induces the cell wall integrity pathway. Genome-wide expression analysis of *A. niger* treated with sub-lethal concentrations of caspofungin resulted in induced expression of 166 genes [20]. These genes are considered to represent the cell wall stress-responsive genes in *A. niger*. To examine whether loss of *tupA* function results in derepression of the cell wall stress-responsive genes we determined the overlap between caspofungin-induced and *tupA*-induced genes. Of the 166 genes induced by caspofungin (CA) only 47 genes (∼28%) were also induced in the *tupA* strain (Table S8). Eighty-six (∼52%) of the CA-induced genes were not differentially expressed in the *tupA* strain. In addition, a considerable number of CA-induced genes (33 genes; ∼20%) had lower expression levels in the *tupA* mutant. Although the overlap is substantial, this indicates that loss of *tupA* function under the growth conditions used here does not simply lead to derepression of cell wall stress-induced genes and suggests that TupA does not function as a repressor of cell wall stress-induced genes under non-stressed conditions.

Interestingly, the two strongest induced cell wall-related genes in the *tupA* mutant, *bgtA* and *exgA*, are not expressed under normal growth conditions. We noticed that these two genes are very highly expressed during sclerotia formation in the *A. niger sclA-1* mutant strain [73], based on RNA analysis extracted from sclerotia (Jørgenson and Ram, unpublished results). In several filamentous fungi, Tup1 functions as a global repressor which regulates genes associated with morphological differentiation, sexual and asexual reproduction, and pathogenicity [31]–[33]. Possibly, TupA of *A. niger* also has a repressing role with respect to the expression of sclerotia-associated genes under conditions in which sclerotia formation is normally repressed.

An additional interesting phenotype of the *tupA* mutant is the secretion of a currently unknown pigment in the medium when grown at high temperatures and with nitrate as a nitrogen source (Fig 1). Both replacement of nitrate by ammonium (Figure 2) as well as the addition of yeast extract and casamino acids to nitrate-containing minimal medium (not shown) reduce pigment production. This suggests that production of the pigment is normally repressed in the presence of nitrate, but that nitrate-controlled repression is lost in the *tupA* mutant. In the *tupA* mutant however, the synthesis of the pigment can still be repressed by more preferred nitrogen sources such as ammonium. In *Penicillium marneffi* the lack of *tupA* also results in pigment production [32], but the effect of nitrogen sources has not been analyzed. Attempts to identify the nature of the pigment secreted in the *tupA* mutant have not been conclusive. It probably has an elemental composition of C10H14O4, but further analysis is required to identify the compound (Kristian F. Nielsen, unpublished results). Intriguingly, the *tup1* deletion strain of *C. albicans* also secretes excessive amounts of a (yellow-green) pigment into the medium [74].

In *S. cerevisiae*, it is well established that Tup1, together with Ssn6 (Cyc8), is involved in carbon repression. In *S. cerevisiae* Mig1p, the repressor responsive to the carbon status of the cell, is known to recruite the Tup1/Ssn6 complex to enable its repressor function [75]. Also in the yeasts *Schizosaccharomyces pombe* and *Candida albicans* Tup1 homologs have been found to be required for carbon repression [76]–[77]. However, in *Aspergillus nidulans* it has been shown that the Tup1 homolog, in this species designated as RcoA, is not involved in carbon repression [78]–[79].

Delmas *et al*. recently proposed a model by which starvation leads to the expression of genes that encode extracellular enzymes [80]. These enzymes are normally repressed by CreA and it was proposed that these enzymes have a sensing role for the presence of alternative substrates. These genes were identified in the study of Delmas et al., because the genes encoding these extracellular enzymes were rapidly induced under carbon starvation conditions. The authors show that the gene encoding cellobiohydrolase (An01g11660; *cbhB*) is induced upon C-starvation in a XlnR-independent way and that the expression is higher in a *creA* mutant strain, strongly suggesting that the induced expression of *cbhB* upon starvation is mediated via carbon catabolite derepression. As *cbhB* (An01g11660) is not higher expressed in the *tupA* mutant, this offers further support for the notion that similar to *A. nidulans tupA* of *A. niger* is not required for carbon catabolite repression.

The expression of the two major extracellular proteases in *A. niger* (*pepA* and *pepB*) has been shown to be under carbon catabolite repression [81]. The involvement of CreA in mediating this repression was shown by [82] as the expression of *pepA* and *pepB* was higher in the *creA* strain under repressing conditions. The latter observation seems to contradict the conclusion the TupA is not mediating carbon repression since *pepA* and *pepB* are highly expressed in the *tupA* mutant (Table S7). To explain these observations, it is important to note that *pepA* and *pepB* are also under nitrogen repression control. The presence of a preferred nitrogen source such as ammonium strongly represses the expression of *pepA* and *pepB* [80]. Additional support for a connection between TupA and nitrogen repression comes from the observation that the formation of the pigment in the *tupA* mutant strain was found to be nitrogen source-dependent and repressed by ammonium.

TupA has been implicated in several fungi to have a role in dimorphism in the transition from yeast to filamentous growth [31]–[33], [83]. The role of Tup1 in fungal dimorphism might well be linked to nitrogen metabolism as nitrogen availability has been shown to be an important factor in fungal dimorphism [84]–[88]. We suggest that the link to nitrogen metabolism and TupA is important to understand the involvement of TupA in developmental processes and dimorphic switches in fungi.

## Supporting Information

Figure S1 Spores of the MA246.1 (*ΔtupA* in RD15.8) were inoculated in MM-glucose containing 10 mM ammonium or 10 mM nitrate at pH3.0 or pH5.7. Pictures were taken after 16 of incubation at 30°C. The fluorescence detected under all conditions shows that *agsA* expression in germinating spores of the *tupA* mutant is not affected by pH or nitrogen source.
(TIF)

Figure S2 Northern blot analysis of selected differentially expressed genes of RNA samples that were used for the microarrays of the wild-type strain (wt) or the *tupA* mutant. Gene identifiers are indicated as well as the gene name (when available). Behind the gene identified the fold change in expression (*tupA* vs wild-type) is given based on the microarray data.
(TIFF)

Table S1 Expression data WT and *tupA* mutant.
(XLS)

Table S2 GO-terms of up-regulated genes in *tupA* mutant.
(XLS)

Table S3 GO-terms of down-regulated genes in *tupA* mutant.
(XLS)

Table S4 TupA up-regulated gene with Fold change >10.
(XLSX)

Table S5 TupA down-regulated gene with Fold change >10.
(XLSX)

Table S6 Expression analysis of all secondary metabolite genes.
(DOCX)

Table S7 Expression analysis of PrtT target genes.
(DOCX)

Table S8 Comparison of caspofungin treated and TupA differentials.
(XLSX)
