# Supplementary figures and images for: The Transcriptional Repressor TupA in Aspergillus niger Is Involved in Controlling Gene Expression Related to Cell Wall Biosynthesis, Development, and Nitrogen Source Availability
Source: PLoS One. 2013 Oct 29;8(10):e78102. doi: 10.1371/journal.pone.0078102 (PMC3812127; doi:10.1371/journal.pone.0078102)

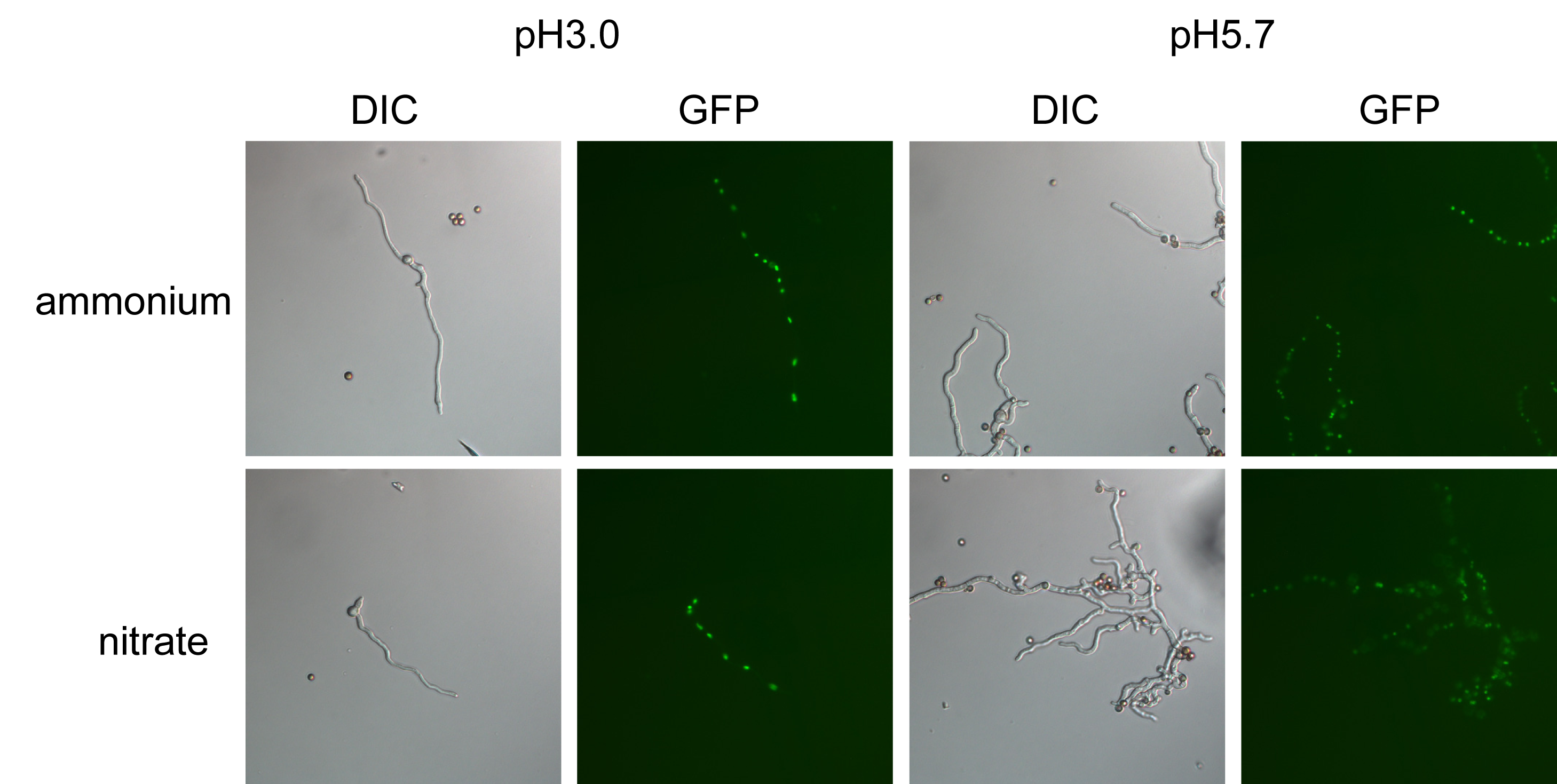

Supplement: Figure S1 — Spores of the MA246.1 (ΔtupA in RD15.8) were inoculated in MM-glucose containing 10 mM ammonium or 10 mM nitrate at pH3.0 or pH5.7. Pictures were taken after 16 of incubation at 30°C. The fluorescence detected under all conditions shows that agsA expression in germinating spores of the tupA mutant is not affected by pH or nitrogen source. (TIF) [file pone.0078102.s001.tif]

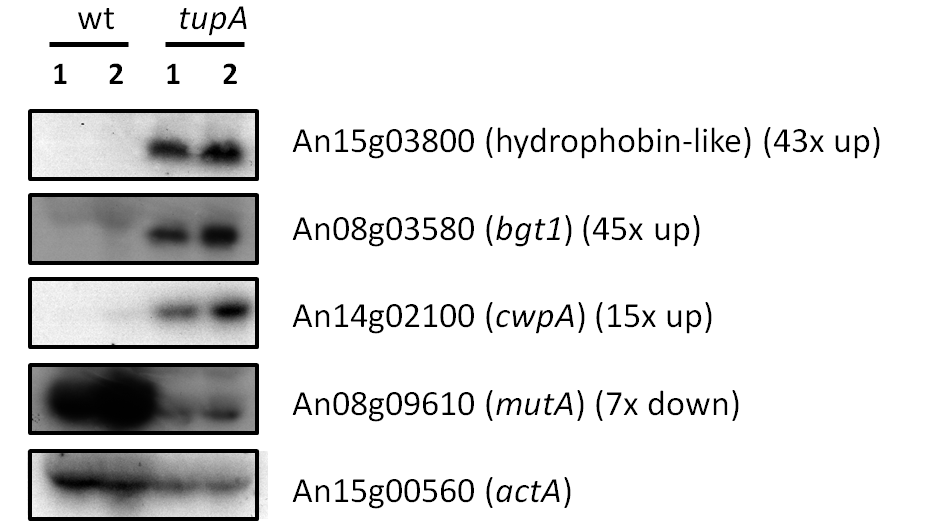

Supplement: Figure S2 — Northern blot analysis of selected differentially expressed genes of RNA samples that were used for the microarrays of the wild-type strain (wt) or the tupA mutant. Gene identifiers are indicated as well as the gene name (when available). Behind the gene identified the fold change in expression (tupA vs wild-type) is given based on the microarray data. (TIFF) [file pone.0078102.s002.tif]
