# Supplementary material for: The Transcriptional Repressor TupA in Aspergillus niger Is Involved in Controlling Gene Expression Related to Cell Wall Biosynthesis, Development, and Nitrogen Source Availability
Source: PLoS One. 2013 Oct 29;8(10):e78102. doi: 10.1371/journal.pone.0078102 (PMC3812127; doi:10.1371/journal.pone.0078102)
Supplement: Table S6 — Expression analysis of all secondary metabolite genes. (DOCX) [file pone.0078102.s008.docx]

Table S6. Genes related to secondary metabolite production (all). Significant differentially expressed genes (P<0.005) are marked yellow. Genes higher expressed in *tupA* mutant are marked red, Genes lower expressed in the *tupA* mutant are marked green.

|  |  | TupA | WT | Tup  ^down^ | P-value | Tup  ^uP^ |
| --- | --- | --- | --- | --- | --- | --- |
|  |  |  |  |  |  |  |
|  |  |  |  |  |  |  |
| **ORF code** | **DSM annotation** | **MT** | **WT** | **FC** | **FDR** | **FC** |
|  |  |  |  |  |  |  |
| An01g01130 | strong similarity to polyketide synthase FUM5 - Gibberella moniliformis | 16 | 17 | 0,9 | 4,32E-01 | 1,1 |
| An01g02030 | similarity to polyketide synthase FUM5 - Gibberella moniliformis | 59 | 63 | 0,9 | 5,65E-01 | 1,1 |
|  |  |  |  |  |  |  |
|  |  |  |  |  |  |  |
| An01g06820 | strong similarity to fatty acid omega-hydroxylase (P450foxy) CYP505 - Fusarium oxysporum | 18 | 20 | 0,9 | 4,16E-01 | 1,1 |
| An01g06830 | similarity to 3-ketosphinganine reductase Tsc10 - Saccharomyces cerevisiae | 20 | 23 | 0,9 | 1,34E-01 | 1,2 |
| An01g06840 | strong similarity to acid-CoA ligase Fat2 - Saccharomyces cerevisiae | 14 | 15 | 0,9 | 2,45E-01 | 1,1 |
| An01g06850 | similarity to 4-hydroxybutyrate dehydrogenase - Alcaligenes eutrophus | 23 | 27 | 0,9 | 1,58E-01 | 1,2 |
| An01g06860 | strong similarity to hypothetical protein Fum9p - Gibberella moniliformis | 20 | 21 | 1,0 | 8,12E-01 | 1,0 |
| An01g06870 | strong similarity to hypothetical protein Fum8p - Gibberella moniliformis | 21 | 22 | 1,0 | 9,05E-01 | 1,0 |
| An01g06880 | similarity to dihydroflavonol 4-reductase BAA12723.1 - Rosa hybrid cultivar | 20 | 23 | 0,9 | 1,82E-01 | 1,1 |
| An01g06890 | similarity to peptide synthase pesA - Metarhizium anisopliae | 15 | 20 | 0,8 | 9,77E-02 | 1,3 |
| An01g06900 | weak similarity to transcription regulator of maltose utilization amyR - Aspergillus oryzae | 17 | 106 | 0,2 | 7,77E-05 | 6,4 |
| An01g06910 | strong similarity to cytochrome P450 CYP94A5 - Nicotiana tabacum | 91 | 257 | 0,4 | 2,60E-05 | 2,8 |
| An01g06920 | strong similarity to multidrug resistance protein ABCC2 - Homo sapiens | 396 | 2265 | 0,2 | 7,99E-07 | 5,7 |
| An01g06930 | strong similarity to polyketide synthase FUM5 - Gibberella moniliformis | 18 | 22 | 0,8 | 1,23E-01 | 1,2 |
| An01g06940 | strong similarity to hypothetical transmembrane transport protein SCC30.17c - Streptomyces coelicolor | 19 | 19 | 1,0 | 8,77E-01 | 1,0 |
| An01g06950 | strong similarity to polyketide synthase FUM5 - Gibberella moniliformis | 20 | 21 | 1,0 | 6,87E-01 | 1,0 |
|  |  |  |  |  |  |  |
| An01g11770 | similarity to peptide synthase enniatin synthase esyn1 - Fusarium scirpi | 16 | 17 | 0,9 | 5,13E-01 | 1,1 |
|  |  |  |  |  |  |  |
| An01g12040 | similarity to polyketide synthase FUM5 - Gibberella moniliformis | 26 | 25 | 1,0 | 8,81E-01 | 1,0 |
|  |  |  |  |  |  |  |
| An01g13170 | strong similarity to hypothetical peptide synthase-like protein CPS1 - Cochliobolus heterostrophus | 74 | 72 | 1,0 | 9,15E-01 | 1,0 |
|  |  |  |  |  |  |  |
| An02g00210 | strong similarity to nonribosomal peptide synthase MxaA - Stigmatella aurantiaca | 1087 | 2062 | 0,5 | 1,56E-04 | 1,9 |
|  |  |  |  |  |  |  |
| An02g00450 | strong similarity to lovastatin diketide synthase lovF - Aspergillus terreus [putative sequencing error] | 48 | 52 | 0,9 | 5,04E-01 | 1,1 |
|  |  |  |  |  |  |  |
| An02g00840 | similarity to nonribosomal peptide synthase MxcG - Stigmatella aurantiaca | 736 | 32 | 23,1 | 6,58E-07 | 0,0 |
|  |  |  |  |  |  |  |
| An02g02300 | weak similarity to nonribosomal peptide synthetase tex1 - Hypocrea virens | 19 | 18 | 1,0 | 8,65E-01 | 1,0 |
|  |  |  |  |  |  |  |
| An02g05070 | strong similarity to d-lysergyl-peptide-synthase PS1 - Claviceps purpurea | 20 | 18 | 1,2 | 1,54E-01 | 0,9 |
| An02g05080 | strong similarity to maackiain detoxification protein MAK1 - Nectria haematococca | 39 | 40 | 1,0 | 8,35E-01 | 1,0 |
| An02g05090 | strong similarity to cytochrome P450 monooxygenase P450I - Gibberella fujikuroi | 24 | 26 | 0,9 | 4,91E-01 | 1,1 |
|  |  |  |  |  |  |  |
| An02g08290 | strong similarity to lovastatin nonaketide synthase lovB - Aspergillus terreus | 50 | 631 | 0,1 | 3,25E-07 | 12,5 |
|  |  |  |  |  |  |  |
| An02g09420 | strong similarity to cytochrome P450 trichodiene oxygenase TRI4 - Fusarium sporotrichioides | 16 | 16 | 1,0 | 9,43E-01 | 1,0 |
| An02g09430 | strong similarity to polyketide synthase FUM5 - Gibberella moniliformis [putative frameshift] | 17 | 19 | 0,9 | 1,50E-01 | 1,1 |
|  |  |  |  |  |  |  |
| An02g10140 | strong similarity to o peptide-polyketide synthase McyG - Microcystis aeruginosa | 20 | 25 | 0,8 | 6,18E-02 | 1,2 |
|  |  |  |  |  |  |  |
| An02g13080 | similarity to N epsilon-(indole-3-acetyl)-L-lysine synthase iaaL - Pseudomonas syringae | 305 | 47 | 6,5 | 4,09E-06 | 0,2 |
| An02g13090 | strong similarity to alanine racemase TOXG - Cochliobolus carbonum | 55 | 31 | 1,8 | 1,34E-02 | 0,6 |
|  |  |  |  |  |  |  |
| An03g00650 | similarity to cyclic peptide AM-toxin synthase AMT - Alternaria alternata | 14 | 15 | 0,9 | 6,08E-01 | 1,1 |
| An03g00660 | similarity to taurine dioxygenase tauD - Escherichia coli | 25 | 42 | 0,6 | 5,84E-04 | 1,7 |
| An03g00670 | similarity to D-nopaline dehydrogenase nos - Agrobacterium tumefaciens | 24 | 23 | 1,0 | 9,76E-01 | 1,0 |
| An03g00680 | strong similarity to multidrug resistance protein fnx1p - Schizosaccharomyces pombe | 25 | 104 | 0,2 | 2,90E-06 | 4,2 |
|  |  |  |  |  |  |  |
| An03g01820 | strong similarity to melanin polyketide synthase PKS - Nodulisporium sp. | 57 | 44 | 1,3 | 5,40E-02 | 0,8 |
|  |  |  |  |  |  |  |
|  |  |  |  |  |  |  |
| An03g03520 | similarity to cyclic peptide AM-toxin synthase AMT - Alternaria alternata | 41 | 343 | 0,1 | 1,15E-06 | 8,4 |
| An03g03530 | similarity to protein fragment SEQ ID NO:9681 from patent EP1033405-A2 - Arabidopsis thaliana | 148 | 1110 | 0,1 | 8,93E-07 | 7,5 |
| An03g03540 | similarity to aerobactin biosynthesis protein iucB - Escherichia coli | 117 | 1441 | 0,1 | 2,87E-06 | 12,3 |
| An03g03550 | strong similarity to carnitine racemase caiD - Escherichia coli | 64 | 1024 | 0,1 | 1,68E-07 | 16,1 |
| An03g03560 | strong similarity to ferrichrome-type siderophore transporter Arn1 - Saccharomyces cerevisiae | 42 | 4167 | 0,0 | 5,23E-08 | 98,8 |
|  |  |  |  |  |  |  |
| siderophoretransporter |  |  |  |  |  |  |
| An03g03620 | strong similarity to multidrug resistance protein atrD - Aspergillus nidulans | 38 | 488 | 0,1 | 7,99E-07 | 12,9 |
|  |  |  |  |  |  |  |
| An03g05130 | similarity to hypothetical dihydrofolate reductase CAB16576.1 - Schizosaccharomyces pombe | 21 | 21 | 1,0 | 9,76E-01 | 1,0 |
| An03g05140 | strong similarity to polyketide synthase PKS1 - Cochliobolus heterostrophus | 16 | 18 | 0,9 | 2,55E-01 | 1,1 |
| An03g05150 | strong similarity to D-mandelate dehydrogenase - Rhodotorula graminis | 21 | 22 | 0,9 | 5,83E-01 | 1,1 |
| An03g05160 | similarity to ferric/cupric reductase Fre2 - Saccharomyces cerevisiae | 31 | 35 | 0,9 | 1,56E-01 | 1,2 |
|  |  |  |  |  |  |  |
| An03g05420 | strong similarity to nitrogen metabolic repression regulator protein hNmrr from patent CN1269419-A - Homo sapiens | 22 | 29 | 0,8 | 1,51E-02 | 1,3 |
| An03g05430 | strong similarity to O-methyltransferase A omtA - Aspergillus parasiticus | 24 | 26 | 0,9 | 3,97E-01 | 1,1 |
| An03g05440 | strong similarity to polyketide synthase alb1 - Aspergillus fumigatus | 15 | 14 | 1,0 | 9,01E-01 | 1,0 |
| An03g05450 | similarity to zeaxanthin epoxidase ABA2 - Nicotiana plumbaginifolia | 21 | 24 | 0,9 | 3,34E-01 | 1,1 |
| An03g05460 | strong similarity to cytochrome P450 monooxygenase avnA - Aspergillus parasiticus | 17 | 18 | 1,0 | 8,77E-01 | 1,0 |
| An03g05470 | strong similarity to hypothetical protein SCD69.03 - Streptomyces coelicolor | 16 | 16 | 1,0 | 8,04E-01 | 1,0 |
| An03g05480 | similarity to O-methyltransferase omtA - Aspergillus parasiticus | 17 | 16 | 1,0 | 8,43E-01 | 1,0 |
| An03g05490 | strong similarity to maleylacetate reductase macA - Rhodococcus opacus | 43 | 52 | 0,8 | 8,40E-02 | 1,2 |
|  |  |  |  |  |  |  |
| An03g05660 | strong similarity to catalase C catC - Aspergillus nidulans | 50 | 55 | 0,9 | 5,03E-01 | 1,1 |
| An03g05670 | strong similarity to aryl-alcohol oxidase precursor aao - Pleurotus pulmonarius | 46 | 39 | 1,2 | 2,26E-01 | 0,9 |
| An03g05680 | strong similarity to peptide synthase mps - Mycobacterium smegmatis | 16 | 19 | 0,8 | 8,04E-02 | 1,2 |
| An03g05990 | strong similarity to O-methyltransferase B omtB - Aspergillus parasiticus | 15 | 15 | 1,0 | 8,58E-01 | 1,0 |
| An03g06000 | similarity to 6-Hydroxy-D-nicotine oxidase 6-HDNO - Arthrobacter oxidans | 23 | 24 | 0,9 | 6,00E-01 | 1,1 |
| An03g06010 | strong similarity to cyclic peptide AM-toxin synthase AMT - Alternaria alternata | 23 | 24 | 1,0 | 6,84E-01 | 1,0 |
|  |  |  |  |  |  |  |
| An03g06370 | weak similarity to transcription activator Cha4 - Saccharomyces cerevisiae | 58 | 60 | 1,0 | 7,19E-01 | 1,0 |
| An03g06380 | strong similarity to polyketide synthase FUM5 - Gibberella moniliformis | 26 | 28 | 0,9 | 5,38E-01 | 1,1 |
| An03g06390 | strong similarity to 3-(3-hydroxyphenyl)propionate hydroxylase MhpA - Comamonas testosteroni | 61 | 92 | 0,7 | 3,30E-03 | 1,5 |
| An03g06400 | similarity to zinc-finger transcription factor amdA - Aspergillus nidulans | 22 | 30 | 0,7 | 7,14E-03 | 1,4 |
| An03g06410 | strong similarity to methyl sterol oxidase Erg25 - Saccharomyces cerevisiae | 37 | 511 | 0,1 | 3,24E-07 | 13,9 |
| An03g06420 | similarity to hypothetical protein encoded by An08g08870 - Aspergillus niger | 33 | 51 | 0,7 | 5,68E-03 | 1,5 |
| An03g06430 | strong similarity to cinnamyl-alcohol dehydrogenase MsaCAD1 - Medicago sativa | 80 | 92 | 0,9 | 1,79E-01 | 1,1 |
| An03g06440 | strong similarity to hypothetical protein EAA64120.1 - Aspergillus nidulans | 23 | 22 | 1,0 | 7,60E-01 | 1,0 |
| An03g06450 | weak similarity to protein SEQ ID NO: 2310 from patent US6562958-B1 - Acinetobacter baumannii | 146 | 135 | 1,1 | 4,53E-01 | 0,9 |
| An03g06460 | strong similarity to hypothetical sterigmatocystin biosynthesis p450 monooxygenase stcB - Aspergillus nidulans | 25 | 26 | 1,0 | 6,86E-01 | 1,0 |
| An03g06470 | weak similarity to MigA - Dictyostelium discoideum | 42 | 44 | 1,0 | 7,17E-01 | 1,0 |
| An03g06480 | strong similarity to Steroid monooxygenase smo - Rhodococcus rhodochrous | 50 | 80 | 0,6 | 1,65E-03 | 1,6 |
| An03g06490 | similarity to heroin esterase her - Rhodococcus sp. | 21 | 24 | 0,9 | 2,19E-01 | 1,1 |
| An03g06500 | strong similarity to zeaxanthin epoxidase ABA2 - Nicotiana plumbaginifolia | 20 | 19 | 1,1 | 4,53E-01 | 0,9 |
| An03g06510 | weak similarity to regulator protein Uga3 - Saccharomyces cerevisiae | 16 | 19 | 0,8 | 1,60E-01 | 1,2 |
|  |  |  |  |  |  |  |
| An04g01140 | strong similarity to 8-amino-7-oxononanoate synthase/KAPA synthase BioF - Kurthia sp. | 18 | 19 | 0,9 | 5,91E-01 | 1,1 |
| An04g01150 | strong similarity to nonribosomal peptide synthase MxaA - Stigmatella aurantiaca | 26 | 27 | 0,9 | 6,10E-01 | 1,1 |
| An04g01160 | strong similarity to hypothetical ABC transporter SPBC15C4.02 - Schizosaccharomyces pombe | 76 | 105 | 0,7 | 4,82E-03 | 1,4 |
|  |  |  |  |  |  |  |
| An04g04330 | strong similarity to 4-coumarate-CoA ligase 4 4CL4 - Glycine max | 99 | 103 | 1,0 | 8,11E-01 | 1,0 |
| An04g04340 | strong similarity to nonaketide synthase lovB - Aspergillus terreus | 25 | 27 | 0,9 | 5,37E-01 | 1,1 |
| An04g04350 | similarity to hypothetical protein encoded by prophage CP-933X Z1925 - Escherichia coli | 33 | 36 | 0,9 | 5,00E-01 | 1,1 |
| An04g04360 | similarity to hypothetical protein 104H10.250 - Neurospora crassa | 18 | 19 | 0,9 | 6,03E-01 | 1,1 |
| An04g04370 | strong similarity to phenylalanine ammonia-lyase Pal - Rhodosporidium toruloides | 12 | 13 | 1,0 | 7,07E-01 | 1,0 |
| An04g04380 | strong similarity to nonribosomal peptide synthase MxaA - Stigmatella aurantiaca | 41 | 42 | 1,0 | 7,88E-01 | 1,0 |
| An04g04390 | similarity to phthalate ester hydrolase pehA - Arthrobacter keyseri | 1717 | 2793 | 0,6 | 8,42E-04 | 1,6 |
|  |  |  |  |  |  |  |
| An04g05420 | strong similarity to alpha-aminoadipate reductase large subunit lys2 - Penicillium chrysogenum | 307 | 366 | 0,8 | 6,62E-02 | 1,2 |
|  |  |  |  |  |  |  |
| An04g06240 | similarity to transcription regulator TRI10 - Fusarium sporotrichioides | 22 | 25 | 0,9 | 2,18E-01 | 1,1 |
| An04g06250 | strong similarity to HC-toxin efflux pump TOXA - Cochliobolus carbonum | 49 | 68 | 0,7 | 1,99E-02 | 1,4 |
| An04g06260 | strong similarity to peptide synthase TycC - Bacillus brevis | 15 | 15 | 1,0 | 8,17E-01 | 1,0 |
| An04g06270 | similarity to hypothetical methyltransferase AAO27746.2 - Fusarium sporotrichioides | 61 | 56 | 1,1 | 3,61E-01 | 0,9 |
|  |  |  |  |  |  |  |
| An04g09500 | strong similarity to hypothetical aldehyde reductase 6 alr6 - Colletotrichum gloeosporioides | 17 | 16 | 1,1 | 5,12E-01 | 0,9 |
| An04g09510 | similarity to hypothetical UbiE/COQ5 family methlytransferase - Caulobacter crescentus | 33 | 36 | 0,9 | 5,36E-01 | 1,1 |
| An04g09520 | strong similarity to O-methyltransferase omtB - Aspergillus flavus | 36 | 33 | 1,1 | 5,11E-01 | 0,9 |
| An04g09530 | strong similarity to melanin polyketide synthase PKS - Nodulisporium sp. | 21 | 21 | 1,0 | 7,59E-01 | 1,0 |
| An04g09540 | similarity to fatty acid omega-hydroxylase cytochrome P-450 CYP4A4 - Oryctolagus cuniculus | 19 | 20 | 1,0 | 7,49E-01 | 1,1 |
| An04g09550 | strong similarity to 4-Hydroxyacetophenone monooxygenase hapE - Pseudomonas fluorescens | 26 | 73 | 0,3 | 6,33E-05 | 2,9 |
|  |  |  |  |  |  |  |
| An04g10030 | strong similarity to polyketide synthase PKS1 - Cochliobolus heterostrophus | 20 | 19 | 1,0 | 7,60E-01 | 1,0 |
|  |  |  |  |  |  |  |
| An04g10100 | similarity to polyketide synthase PKS17 - Botryotinia fuckeliana | 18 | 18 | 1,0 | 9,75E-01 | 1,0 |
|  |  |  |  |  |  |  |
| An05g01060 | strong similarity to HC-toxin synthase HTS1 - Cochliobolus carbonum | 24 | 150 | 0,2 | 3,09E-06 | 6,4 |
| An05g01070 | strong similarity to 7-aminocholesterol resistance protein Rta1 - Saccharomyces cerevisiae | 484 | 4407 | 0,1 | 1,94E-06 | 9,1 |
| An05g01080 | weak similarity to potassium channel protein AKT1 - Arabidopsis thaliana | 20 | 29 | 0,7 | 3,16E-03 | 1,5 |
| An05g01090 | questionable ORF | 21 | 23 | 0,9 | 5,27E-01 | 1,1 |
| An05g01100 | strong similarity to HC-toxin biosynthesis protein TOXF - Cochliobolus carbonum | 27 | 42 | 0,6 | 3,46E-03 | 1,6 |
| An05g01110 | strong similarity to cadmium resistance protein Ycf1 - Saccharomyces cerevisiae | 25 | 26 | 0,9 | 7,03E-01 | 1,1 |
| An05g01120 | strong similarity to hypothetical cytochrome P450 monooxygenase TRI11 - Fusarium sporotrichioides | 22 | 21 | 1,0 | 8,81E-01 | 1,0 |
|  |  |  |  |  |  |  |
| An05g00220 | strong similarity to L-ornithine N5-hydroxylase psbA - Pseudomonas sp. | 96 | 1399 | 0,1 | 1,68E-07 | 14,5 |
| An06g01300 | strong similarity to cyclic peptide AM-toxin synthase AMT - Alternaria alternata | 92 | 153 | 0,6 | 7,75E-04 | 1,7 |
| An06g01310 | hypothetical protein | 11 | 12 | 0,9 | 5,75E-01 | 1,1 |
| An06g01320 | strong similarity to feruloyl-CoA synthase fcs - Amycolatopsis sp. | 31 | 102 | 0,3 | 9,11E-06 | 3,3 |
| An07g01030 | strong similarity to polyketide synthase FUM5 - Gibberella moniliformis | 34 | 38 | 0,9 | 4,12E-01 | 1,1 |
| An07g01040 | weak similarity to polyketide synthase Fum5 - Gibberella moniliformis | 25 | 26 | 1,0 | 8,18E-01 | 1,0 |
| An08g02290 | strong similarity to fluconazole resistance protein FLU1 - Candida albicans | 48 | 51 | 1,0 | 6,39E-01 | 1,1 |
| An08g02300 | weak similarity to enniatin synthase - Fusarium scirpi [truncated ORF] | 1065 | 553 | 1,9 | 2,10E-04 | 0,5 |
|  |  |  |  |  |  |  |
| An08g02310 | similarity to HC-toxin non-ribosomal peptide synthase HTS1 - Cochliobolus carbonum [truncated ORF] | 77 | 55 | 1,4 | 6,00E-03 | 0,7 |
| An08g02320 | questionable ORF | 46 | 39 | 1,2 | 8,91E-02 | 0,9 |
| An08g02330 | strong similarity to multidrug resistance protein MLP-2 - Rattus norvegicus | 899 | 247 | 3,6 | 2,45E-05 | 0,3 |
|  |  |  |  |  |  |  |
| An08g03730 | similarity to gibberellin 7-oxidase - Cucurbita maxima | 264 | 32 | 8,2 | 1,19E-05 | 0,1 |
| An08g03740 | strong similarity to enoyl reductase of the lovastatin biosynthesis lovC - Aspergillus terreus | 131 | 50 | 2,6 | 5,76E-04 | 0,4 |
| An08g03750 | strong similarity to fluconazole resistance protein FLU1 - Candida albicans | 366 | 49 | 7,4 | 2,51E-06 | 0,1 |
| An08g03760 | similarity to hypothetical protein Rv3472 - Mycobacterium tuberculosis | 2763 | 411 | 6,7 | 2,10E-06 | 0,1 |
| An08g03770 | weak similarity to mucin MUC5AC - Homo sapiens Gal4-like TF | 607 | 19 | 31,4 | 7,99E-07 | 0,0 |
| An08g03780 | strong similarity to cytochrome P450 monooxygenase TRI11 - Fusarium sporotrichioides | 96 | 32 | 3,0 | 1,59E-04 | 0,3 |
| An08g03790 | strong similarity to lovastatin nonaketide synthase lovB - Aspergillus terreus | 26 | 28 | 1,0 | 6,81E-01 | 1,0 |
| An08g03800 | similarity to saframycin Mx1 synthase safA - Myxococcus xanthus [truncated ORF] | 52 | 41 | 1,3 | 5,65E-02 | 0,8 |
| An08g03810 | similarity to hypothetical protein encoded by An02g00870 - Aspergillus niger | 20 | 19 | 1,1 | 4,38E-01 | 0,9 |
| An08g03820 | strong similarity to enoyl reductase of the lovastatin biosynthesis lovC - Aspergillus terreus | 18 | 20 | 0,9 | 3,48E-01 | 1,1 |
|  |  |  |  |  |  |  |
| An08g04820 | similarity to saframycin Mx1 synthase safA - Myxococcus xanthus | 33 | 38 | 0,9 | 3,47E-01 | 1,1 |
|  |  |  |  |  |  |  |
| An08g09220 | similarity to multifunctional peptide synthetase of the nostopeptolide biosynthetis NosD - Nostoc sp. | 43 | 49 | 0,9 | 1,52E-01 | 1,1 |
| An08g09230 | strong similarity to isotrichodermin C-15 hydroxylase Tri11 - Fusarium sporotrichioides | 28 | 35 | 0,8 | 3,76E-02 | 1,2 |
|  |  |  |  |  |  |  |
| An08g10860 | strong similarity to fatty acid synthase beta subunit fasB - Aspergillus nidulans | 14 | 15 | 1,0 | 7,86E-01 | 1,0 |
| An08g10870 | strong similarity to 2-methylcitrate dehydratase PrpD - Salmonella typhimurium | 19 | 18 | 1,1 | 4,95E-01 | 0,9 |
| An08g10880 | strong similarity to regulator protein of lovastatin biosynthesis gene cluster encoded by ORF13 - Aspergillus terreus | 19 | 21 | 0,9 | 4,54E-01 | 1,1 |
| An08g10890 | questionable ORF | 23 | 28 | 0,8 | 5,28E-02 | 1,2 |
| An08g10900 | hypothetical protein | 32 | 31 | 1,0 | 7,68E-01 | 1,0 |
| An08g10910 | questionable ORF | 18 | 18 | 1,0 | 7,94E-01 | 1,0 |
| An08g10920 | strong similarity to citrate synthase YKPSCA - Pseudomonas aeruginosa | 25 | 32 | 0,8 | 3,69E-02 | 1,3 |
| An08g10930 | strong similarity to fatty acid synthase alpha subunit Fas2 - Saccharomyces cerevisiae | 21 | 22 | 0,9 | 5,36E-01 | 1,1 |
|  |  |  |  |  |  |  |
| An09g00520 | similarity to tyrocidine synthase 2 tycB - Brevibacillus brevis | 15 | 17 | 0,9 | 4,22E-01 | 1,1 |
| An09g00530 | similarity to salicylate hydroxylase nahW - Pseudomonas stutzeri | 50 | 50 | 1,0 | 9,44E-01 | 1,0 |
| An09g00540 | hypothetical protein | 14 | 14 | 1,0 | 9,95E-01 | 1,0 |
| An09g00550 | similarity to multidrug resistance protein fnx1p - Schizosaccharomyces pombe | 20 | 20 | 1,0 | 9,66E-01 | 1,0 |
| An09g00560 | similarity to mitochondrial 25-hydroxyvitamin D3 24-hydroxylase cP450cc24 - Gallus gallus | 55 | 47 | 1,2 | 1,26E-01 | 0,9 |
|  |  |  |  |  |  |  |
| An09g01260 | similarity to oxidoreductase OXRD-1 from patent WO200071679-A2 - Homo sapiens | 21 | 23 | 0,9 | 4,45E-01 | 1,1 |
| An09g01270 | strong similarity to n-alkane inducible cytochrome P450 protein ALK1 - Yarrowia lipolytica | 28 | 29 | 1,0 | 8,98E-01 | 1,0 |
| An09g01280 | questionable ORF | 21 | 21 | 1,0 | 9,97E-01 | 1,0 |
| An09g01290 | strong similarity to polyketide synthase FUM5 - Gibberella moniliformis | 24 | 25 | 1,0 | 6,93E-01 | 1,0 |
| An09g01300 | weak similarity to protein fragment SEQ ID NO:2141 from patent EP1033405-A2 - Arabidopsis thaliana | 15 | 15 | 1,0 | 8,23E-01 | 1,0 |
| An09g01310 | hypothetical protein | 18 | 17 | 1,0 | 6,75E-01 | 1,0 |
| An09g01320 | strong similarity to isoamyl alcohol oxidase mreA - Aspergillus oryzae | 18 | 20 | 0,9 | 3,90E-01 | 1,1 |
| An09g01330 | strong similarity to lanosterol synthase Erg7 - Saccharomyces cerevisiae | 30 | 33 | 0,9 | 4,23E-01 | 1,1 |
| An09g01340 | similarity to leukotriene B(4) omega-hydroxylase cytochrome P450 4F2 CYP4F2 - Homo sapiens | 45 | 46 | 1,0 | 8,56E-01 | 1,0 |
| An09g01350 | questionable ORF | 13 | 13 | 1,0 | 8,36E-01 | 1,0 |
| An09g01370 | strong similarity to lignostilbene-alpha,beta-dioxygenase LSD - Pseudomonas paucimobilis | 23 | 25 | 0,9 | 5,01E-01 | 1,1 |
| An09g01380 | strong similarity to vanillyl-alcohol oxidase vaoA - Penicillium simplicissimum | 37 | 37 | 1,0 | 9,62E-01 | 1,0 |
|  |  |  |  |  |  |  |
| An09g01690 | strong similarity to HC-toxin synthase HTS1 - Cochliobolus carbonum | 15 | 16 | 0,9 | 5,94E-01 | 1,1 |
| An09g01700 | strong similarity to multidrug resistance protein MRP1 - Homo sapiens | 21 | 25 | 0,8 | 1,62E-01 | 1,2 |
| An09g01710 | similarity to epoxide hydrolase from patent EP879890-A - grobacterium radiobacter | 342 | 198 | 1,7 | 1,01E-03 | 0,6 |
| An09g01730 | hypothetical protein | 23 | 27 | 0,8 | 1,52E-01 | 1,2 |
| An09g01740 | strong similarity to beta chain of fatty-acyl-CoA synthase Fas1 - Saccharomyces cerevisiae [truncated ORF] | 25 | 24 | 1,0 | 7,32E-01 | 1,0 |
| An09g01750 | strong similarity to fatty acid synthase beta subunit fasB - Aspergillus nidulans [truncated ORF] | 22 | 22 | 1,0 | 9,98E-01 | 1,0 |
|  |  |  |  |  |  |  |
| An09g01800 | strong similarity to trichothecene 3-O-acetyltransferase TRI101 - Fusarium sporotrichioides | 25 | 24 | 1,0 | 7,65E-01 | 1,0 |
| An09g01810 | strong similarity to ketoreductase from patent EP918090-A - Saccharomyces cerevisiae | 34 | 34 | 1,0 | 9,93E-01 | 1,0 |
| An09g01820 | strong similarity to 4-coumarate-CoA ligase 4CL - Arabidopsis thaliana | 50 | 81 | 0,6 | 2,34E-03 | 1,6 |
| An09g01830 | similarity to 6-hydroxy-D-nicotine oxidase 6-HDNO - Arthrobacter oxidans | 26 | 29 | 0,9 | 2,28E-01 | 1,1 |
| An09g01840 | strong similarity to salicylate hydroxylase nahG - Pseudomonas putida | 49 | 62 | 0,8 | 6,72E-02 | 1,3 |
| An09g01850 | strong similarity to benzoate 4-monooxygenase cytochrome P450 53 bphA - Aspergillus niger | 16 | 16 | 1,0 | 8,90E-01 | 1,0 |
| An09g01860 | strong similarity to polyketide synthase wA - Aspergillus nidulans | 40 | 41 | 1,0 | 8,31E-01 | 1,0 |
| An09g01870 | similarity to hypothetical binuclear zinc transcription factor PRF - Nectria haematococca | 37 | 44 | 0,8 | 8,57E-02 | 1,2 |
| An09g01880 | strong similarity to enoyl reductase of the lovastatin biosynthesis lovC - Aspergillus terreus | 18 | 22 | 0,8 | 1,27E-01 | 1,2 |
|  |  |  |  |  |  |  |
| An09g01910 | similarity to tetracyclin resistance protein tetA - Agrobacterium tumefaciens | 22 | 26 | 0,8 | 3,78E-02 | 1,2 |
| An09g01920 | similarity to 6-hydroxy-D-nicotine oxidase 6-HDNO - Arthrobacter oxidans | 24 | 24 | 1,0 | 8,90E-01 | 1,0 |
|  |  |  |  |  |  |  |
| An09g01930 | strong similarity to lovastatin diketide synthase lovF - Aspergillus terreus | 26 | 23 | 1,1 | 3,50E-01 | 0,9 |
| An09g01940 | strong similarity to 7-aminocholesterol resistance protein Rta1 - Saccharomyces cerevisiae | 16 | 16 | 1,0 | 9,78E-01 | 1,0 |
| An09g01950 | strong similarity to cytochrome P450 monooxygenase TRI11 - Fusarium sporotrichioides | 38 | 48 | 0,8 | 1,45E-01 | 1,2 |
| An09g01960 | hypothetical protein | 14 | 15 | 0,9 | 5,42E-01 | 1,1 |
| An09g01970 | similarity to oxidoreductase from patent WO0100844 - Corynebacterium glutamicum | 32 | 31 | 1,0 | 8,72E-01 | 1,0 |
| An09g01980 | hypothetical protein | 14 | 15 | 1,0 | 8,19E-01 | 1,0 |
| An09g01990 | strong similarity to branched-chain amino acid aminotransferase bcaT - Lactococcus lactis | 24 | 24 | 1,0 | 9,44E-01 | 1,0 |
| An09g02000 | strong similarity to cytochrome P450 - Rhodotorula minuta | 25 | 25 | 1,0 | 8,65E-01 | 1,0 |
| An09g02010 | strong similarity to fatty acid synthase alpha subunit fasA - Aspergillus nidulans | 18 | 20 | 0,9 | 5,57E-01 | 1,1 |
|  |  |  |  |  |  |  |
| An09g02590 | strong similarity to cytochrome P450 monooxygenase stcS - Aspergillus nidulans | 32 | 33 | 1,0 | 8,58E-01 | 1,0 |
| An09g02600 | hypothetical protein | 20 | 21 | 1,0 | 9,05E-01 | 1,0 |
| An09g02610 | similarity to trichodiene synthase - Gibberella pulicaris | 20 | 22 | 0,9 | 6,87E-01 | 1,1 |
| An09g02620 | similarity to transcription factor ntf1p - Schizosaccharomyces pombe | 46 | 45 | 1,0 | 7,74E-01 | 1,0 |
|  |  |  |  |  |  |  |
| An09g05060 | strong similarity to transcription repressor Rdr1 - Saccharomyces cerevisiae | 33 | 38 | 0,9 | 1,94E-01 | 1,1 |
| An09g05070 | strong similarity to fluconazole resistance protein FLU1 - Candida albicans | 31 | 33 | 0,9 | 5,49E-01 | 1,1 |
| An09g05080 | similarity to aromatic aminotransferase I Aro8 - Saccharomyces cerevisiae | 246 | 230 | 1,1 | 6,54E-01 | 0,9 |
| An09g05100 | similarity to Impact - Mus musculus | 35 | 37 | 1,0 | 7,92E-01 | 1,1 |
| An09g05110 | strong similarity to peptide-polyketide synthase McyG - Microcystis aeruginosa | 46 | 44 | 1,0 | 8,48E-01 | 1,0 |
| An09g05120 | similarity to lipase lip1 - Geotrichum candidum | 23 | 24 | 1,0 | 7,33E-01 | 1,1 |
| An09g05130 | similarity to monophenol monooxygenase melC2 - Streptomyces antibioticus | 17 | 17 | 1,0 | 9,99E-01 | 1,0 |
| An09g05340 | strong similarity to polyketide synthase FUM5 - Gibberella moniliformis | 25 | 25 | 1,0 | 9,74E-01 | 1,0 |
| An09g05350 | strong similarity to mature penicillin V amidohydrolase PVA from patent US5516679-A - Fusarium oxysporum | 65 | 72 | 0,9 | 3,57E-01 | 1,1 |
| An09g05720 | hypothetical protein | 18 | 18 | 1,0 | 9,93E-01 | 1,0 |
| An09g05730 | strong similarity to polyketide synthase alb1 - Aspergillus fumigatus | 54 | 128 | 0,4 | 3,94E-04 | 2,4 |
| An09g05740 | hypothetical protein | 37 | 41 | 0,9 | 3,81E-01 | 1,1 |
| An09g05750 | hypothetical protein | 23 | 23 | 1,0 | 9,36E-01 | 1,0 |
|  |  |  |  |  |  |  |
| An10g00110 | strong similarity to O-methylsterigmatocystin oxidoreductase ordA - Aspergillus parasiticus | 46 | 42 | 1,1 | 6,08E-01 | 0,9 |
| An10g00120 | similarity to 2,3-dihydroxybenzoic acid decarboxylase from patent WO9909048-A1 - Aspergillus niger | 25 | 25 | 1,0 | 9,14E-01 | 1,0 |
| An10g00130 | strong similarity to para-hydroxybenzoate--polyprenyltransferase ppt1p - Schizosaccharomyces pombe | 20 | 23 | 0,9 | 1,58E-01 | 1,2 |
| An10g00140 | strong similarity to 6-methylsalicylic acid synthase atX - Aspergillus terreus | 14 | 15 | 1,0 | 8,05E-01 | 1,0 |
| An10g00150 | strong similarity to cytochrome P450 monooxygenase TRI4 - Myrothecium roridum | 17 | 18 | 1,0 | 8,33E-01 | 1,0 |
|  |  |  |  |  |  |  |
| An10g00620 | strong similarity to hypothetical branched-chain amino acid aminotransferase ToxF - Cochliobolus carbonum | 23 | 29 | 0,8 | 9,38E-02 | 1,2 |
| An10g00630 | strong similarity to alpha subunit of the fatty acid synthase fasA - Aspergillus nidulans | 24 | 32 | 0,7 | 1,63E-02 | 1,3 |
| An10g00640 | hypothetical protein | 58 | 64 | 0,9 | 3,81E-01 | 1,1 |
| An10g00650 | strong similarity to fatty acid synthase beta subunit fasB - Aspergillus nidulans | 16 | 17 | 0,9 | 3,97E-01 | 1,1 |
| An10g00660 | strong similarity to cytochrome P450 monooxygenase TRI11 - Fusarium sporotrichioides | 35 | 36 | 1,0 | 6,94E-01 | 1,0 |
|  |  |  |  |  |  |  |
| An11g00050 | strong similarity to enniatin synthase esyn1 - Fusarium scirpi | 26 | 39 | 0,7 | 3,46E-03 | 1,5 |
| An11g00060 | similarity to integral membrane protein PTH11 - Magnaporthe grisea | 982 | 23 | 43,1 | 7,97E-08 | 0,0 |
| An11g00070 | strong similarity to O-methyltransferase B omtB - Aspergillus parasiticus | 251 | 47 | 5,4 | 1,72E-06 | 0,2 |
| An11g00080 | weak similarity to integral membrane protein PTH11 - Magnaporthe grisea | 21 | 23 | 0,9 | 4,89E-01 | 1,1 |
| An11g00090 | similarity to hypothetical membrane protein pth - Blumeria graminis | 60 | 49 | 1,2 | 3,81E-02 | 0,8 |
|  |  |  |  |  |  |  |
| An11g00230 | strong similarity to 6-hydroxy-d-nicotine oxidase 6-HDNO - Arthrobacter oxidans | 27 | 29 | 0,9 | 6,00E-01 | 1,1 |
| An11g00240 | weak similarity to 2-hydroxyisoflavone reductase IRL - Zea mays | 21 | 22 | 1,0 | 7,58E-01 | 1,0 |
| An11g00250 | strong similarity to lovastatin diketide synthase lovF - Aspergillus terreus | 22 | 27 | 0,8 | 1,11E-01 | 1,2 |
| An11g00260 | strong similarity to monocarboxylate transporter MCT3 - Homo sapiens | 23 | 23 | 1,0 | 9,90E-01 | 1,0 |
| An11g00270 | strong similarity to lanosterol 14 alpha-demethylase CYP51 - Homo sapiens | 19 | 20 | 1,0 | 8,70E-01 | 1,0 |
| An11g00280 | strong similarity to O-methyltransferase omtB - Aspergillus flavus | 29 | 29 | 1,0 | 9,08E-01 | 1,0 |
|  |  |  |  |  |  |  |
| An11g00320 | similarity to pristinamycin I synthase 3 SnbDE - Streptomyces pristinaespiralis | 20 | 20 | 1,0 | 8,21E-01 | 1,0 |
| An11g00330 | strong similarity to hypothetical protein encoded by An07g00480 - Aspergillus niger | 83 | 92 | 0,9 | 4,08E-01 | 1,1 |
| An11g00340 | questionable ORF | 13 | 13 | 1,0 | 9,73E-01 | 1,0 |
| An11g00350 | strong similarity to berberine bridge enzyme BBE - Papaver somniferum | 20 | 21 | 0,9 | 6,11E-01 | 1,1 |
|  |  |  |  |  |  |  |
| An11g03920 | strong similarity to lovastatin diketide synthase lovF - Aspergillus terreus | 52 | 55 | 0,9 | 6,16E-01 | 1,1 |
| An11g03930 | similarity to hypothetical protein encoded by An15g02140 - Aspergillus niger | 27 | 24 | 1,1 | 2,49E-01 | 0,9 |
| An11g03940 | strong similarity to bifunctional cytochrome P450rm - Rhodotorula minuta | 108 | 86 | 1,3 | 5,24E-02 | 0,8 |
| An11g03950 | similarity to hypothetical protein required for biosynthesis of the host-specific AK-toxin Akt2 - Alternaria alternata | 185 | 87 | 2,1 | 7,52E-05 | 0,5 |
| An11g03960 | similarity to integral membrane protein PTH11 - Magnaporthe grisea | 48 | 32 | 1,5 | 1,95E-03 | 0,7 |
|  |  |  |  |  |  |  |
| An11g04250 | similarity to aminoadipate reductase enzyme lys2 - Acremonium chrysogenum | 16 | 16 | 1,0 | 8,97E-01 | 1,0 |
| An11g04260 | weak similarity to dihydrofolate reductase dfr1p - Schizosaccharomyces pombe | 26 | 28 | 0,9 | 6,49E-01 | 1,1 |
| An11g04270 | strong similarity to enoyl reductase of the lovastatin biosynthesis lovC - Aspergillus terreus | 18 | 19 | 0,9 | 6,48E-01 | 1,1 |
| An11g04280 | strong similarity to polyketide synthase PKS1 - Cochliobolus heterostrophus | 25 | 26 | 1,0 | 7,85E-01 | 1,0 |
|  |  |  |  |  |  |  |
| An11g05550 | similarity to hypothetical major facilitator transporter Mfs1.1 - Coprinus cinereus | 36 | 36 | 1,0 | 9,98E-01 | 1,0 |
| An11g05560 | similarity to 8-amino-7-oxononanoate synthase bioF - Bacillus sphaericus | 20 | 19 | 1,0 | 7,57E-01 | 1,0 |
| An11g05570 | strong similarity to polyketide synthase FUM5 - Gibberella moniliformis | 16 | 18 | 0,9 | 2,62E-01 | 1,1 |
|  |  |  |  |  |  |  |
| An11g05930 | strong similarity to allantoate permease Dal5 - Saccharomyces cerevisiae | 25 | 29 | 0,9 | 1,63E-01 | 1,2 |
| An11g05940 | strong similarity to polyketide synthase PKS1 - Cochliobolus heterostrophus | 31 | 31 | 1,0 | 9,50E-01 | 1,0 |
| An11g05950 | weak similarity to dihydrofolate reductase dfr1p - Schizosaccharomyces pombe | 18 | 17 | 1,0 | 7,52E-01 | 1,0 |
| An11g05960 | strong similarity to lovastatin diketide synthase lovF - Aspergillus terreus | 18 | 19 | 0,9 | 7,14E-01 | 1,1 |
|  |  |  |  |  |  |  |
| An11g06430 | similarity to integral membrane protein PTH11 - Magnaporthe grisea | 25 | 25 | 1,0 | 9,55E-01 | 1,0 |
| An11g06440 | strong similarity to enoyl reductase of the lovastatin biosynthesis lovC - Aspergillus terreus | 19 | 19 | 1,0 | 9,95E-01 | 1,0 |
| An11g06450 | strong similarity to hypothetical protein encoded by An02g08300 - Aspergillus niger | 19 | 20 | 1,0 | 7,37E-01 | 1,0 |
|  |  |  |  |  |  |  |
| An11g07290 | similarity to spectinomycin adenylyltransferase spc - Staphylococcus aureus | 270 | 303 | 0,9 | 2,63E-01 | 1,1 |
| An11g07300 | similarity to fluconazole resistance protein FLU1 - Candida albicans [truncated ORF] | 47 | 59 | 0,8 | 2,45E-02 | 1,3 |
| An11g07310 | strong similarity to polyketide synthase PKS1 - Colletotrichum lagenarium | 33 | 33 | 1,0 | 9,70E-01 | 1,0 |
| An11g07320 | similarity to glyoxalase II Glo2 - Saccharomyces cerevisiae | 40 | 41 | 1,0 | 9,26E-01 | 1,0 |
| An11g07330 | similarity to 6-hydroxynicotinic acid mono-oxygenase 6-HNAMO from patent JP09121864-A - Pseudomonas fluorescens | 57 | 54 | 1,0 | 7,40E-01 | 1,0 |
| An11g07340 | strong similarity to hypothetical O-methyl transferase EncK - Streptomyces maritimus | 20 | 19 | 1,1 | 6,33E-01 | 0,9 |
| An11g07350 | similarity to transcription regulator amdR - Aspergillus oryzae | 104 | 86 | 1,2 | 1,11E-01 | 0,8 |
| An11g07360 | hypothetical protein | 23 | 23 | 1,0 | 9,65E-01 | 1,0 |
| An11g07370 | questionable ORF | 37 | 44 | 0,8 | 7,76E-02 | 1,2 |
| An11g07380 | similarity to phenazine biosynthesis oxidoreductase phzF - Pseudomonas fluorescens | 341 | 297 | 1,1 | 3,90E-01 | 0,9 |
|  |  |  |  |  |  |  |
| An11g09710 | strong similarity to 4-coumarate-CoA ligase - Populus tremuloides | 132 | 120 | 1,1 | 6,95E-01 | 0,9 |
|  |  |  |  |  |  |  |
| An11g09720 | strong similarity to polyketide synthase PKS1 - Cochliobolus heterostrophus | 46 | 34 | 1,4 | 2,74E-02 | 0,7 |
|  |  |  |  |  |  |  |
| An12g01980 | strong similarity to fatty acid synthase alpha subunit Fas2 - Saccharomyces cerevisiae | 15 | 15 | 1,0 | 9,69E-01 | 1,0 |
| An12g01990 | strong similarity to fatty-acyl-CoA synthase beta chain fas1p - Schizosaccharomyces pombe | 40 | 45 | 0,9 | 3,60E-01 | 1,1 |
| An12g02000 | strong similarity to salicylate hydroxylase nahG - Pseudomonas putida [putative frameshift] | 33 | 36 | 0,9 | 5,10E-01 | 1,1 |
| An12g02010 | weak similarity to hypothetical protein At2g22660 - Arabidopsis thaliana | 29 | 31 | 1,0 | 6,42E-01 | 1,1 |
| An12g02020 | strong similarity to trichothecene 3-O-acetyltransferase TRI101 - Fusarium sporotrichioides | 33 | 33 | 1,0 | 9,80E-01 | 1,0 |
| An12g02030 | weak similarity to hypothetical ATP-binding ABC transporter protein - Deinococcus radiodurans | 46 | 53 | 0,9 | 1,61E-01 | 1,1 |
| An12g02040 | similarity to acetate regulatory DNA binding protein facB - Aspergillus niger | 17 | 17 | 1,0 | 8,56E-01 | 1,0 |
| An12g02050 | strong similarity to polyketide synthase wA - Aspergillus nidulans [putative frameshift] | 20 | 20 | 1,0 | 8,76E-01 | 1,0 |
| An12g02060 | strong similarity to hypothetical protein encoded by An03g02680 - Aspergillus niger | 60 | 261 | 0,2 | 2,99E-06 | 4,3 |
| An12g02070 | similarity to ribonuclease T1 precursor rntA - Aspergillus oryzae | 58 | 139 | 0,4 | 1,43E-04 | 2,4 |
| An12g02080 | strong similarity to cytochrome P450 monooxygenase stcS - Aspergillus nidulans [putative frameshift] | 359 | 935 | 0,4 | 6,84E-05 | 2,6 |
|  |  |  |  |  |  |  |
| An12g02640 | strong similarity to clavin biosynthesis gene orfup1 from patent WO9833896-A2 - Streptomyces clavuligerus | 29 | 37 | 0,8 | 2,86E-02 | 1,2 |
| An12g02650 | similarity to 24-sterol C-methyltransferase ESMT1 - Zea mays | 40 | 456 | 0,1 | 9,96E-07 | 11,4 |
| An12g02660 | hypothetical protein | 24 | 248 | 0,1 | 1,64E-06 | 10,2 |
| An12g02670 | strong similarity to polyketide synthase FUM5 - Gibberella fujikuroi | 27 | 28 | 1,0 | 7,33E-01 | 1,1 |
| An12g02680 | weak similarity to hypothetical protein encoded by An02g12900 - Aspergillus niger | 22 | 21 | 1,0 | 8,68E-01 | 1,0 |
| An12g02690 | weak similarity to cytochrome b5 CB5 - Oryza sativa | 13 | 15 | 0,9 | 2,59E-01 | 1,1 |
| An12g02700 | strong similarity to gluconate 5-dehydrogenase GNO - Gluconobacter oxydans | 31 | 32 | 1,0 | 8,90E-01 | 1,0 |
| An12g02710 | similarity to cercosporin transporter CFP - Cercospora kikuchii | 22 | 22 | 1,0 | 9,83E-01 | 1,0 |
| An12g02720 | similarity to hypothetical protein C25G4.2 - Caenorhabditis elegans | 36 | 39 | 0,9 | 5,14E-01 | 1,1 |
| An12g02730 | strong similarity to polyketide synthase PKS1 - Cochliobolus heterostrophus | 24 | 25 | 1,0 | 6,30E-01 | 1,1 |
| An12g02740 | weak similarity to ATP-dependent proteinase Clp from patent WO9743303-A1 - Streptococcus pneumoniae | 20 | 22 | 0,9 | 2,86E-01 | 1,1 |
| An12g02750 | similarity to FK520 biosynthetic gene cluster polyketide synthase fkbB - Streptomyces hygroscopicus | 16 | 14 | 1,2 | 1,01E-01 | 0,8 |
| An12g02760 | similarity to hypothetical protein BAB49075.1 - Mesorhizobium loti | 21 | 20 | 1,1 | 4,87E-01 | 0,9 |
| An12g02770 | similarity to beta transducin-like protein het-e1 - Podospora anserina | 25 | 26 | 1,0 | 7,53E-01 | 1,0 |
| An12g02780 | strong similarity to hypothetical protein AAO55050.1 - Pseudomonas syringae | 14 | 15 | 0,9 | 4,23E-01 | 1,1 |
| An12g02790 | strong similarity to phenylcoumaran benzylic ether reductase pcbera - Populus trichocarpa | 18 | 17 | 1,0 | 7,62E-01 | 1,0 |
| An12g02800 | strong similarity to polyamine transport protein Tpo1 - Saccharomyces cerevisiae | 20 | 18 | 1,1 | 2,45E-01 | 0,9 |
| An12g02810 | strong similarity to O-methyltransferase omtB - Aspergillus flavus | 22 | 42 | 0,5 | 1,64E-03 | 1,9 |
| An12g02820 | strong similarity to fluconazole resistance protein FLU1 - Candida albicans | 52 | 55 | 0,9 | 6,23E-01 | 1,1 |
| An12g02830 | weak similarity to hypothetical methyltransferase adpE - Anabaena sp. | 55 | 101 | 0,5 | 1,50E-03 | 1,8 |
| An12g02840 | strong similarity to d-lysergyl-peptide-synthase PS1 - Claviceps purpurea | 103 | 110 | 0,9 | 5,35E-01 | 1,1 |
| An12g02850 | strong similarity to sulphydryl oxidase Sox from patent EP565172-A1 - Aspergillus niger | 18 | 18 | 1,0 | 9,32E-01 | 1,0 |
| An12g02860 | strong similarity to hypothetical protein of the lovastatin biosynthesis gene cluster - Aspergillus terreus | 17 | 20 | 0,9 | 2,44E-01 | 1,1 |
|  |  |  |  |  |  |  |
|  |  |  |  |  |  |  |
| An12g03950 | strong similarity to hypothetical protein npgA - Aspergillus nidulans | 92 | 81 | 1,1 | 2,50E-01 | 0,9 |
|  |  |  |  |  |  |  |
| An12g07050 | weak similarity to dihydrofolate reductase dfr1p - Schizosaccharomyces pombe | 41 | 19 | 2,2 | 1,36E-04 | 0,5 |
| An12g07060 | similarity to hypothetical protein YMR222c - Saccharomyces cerevisiae | 43 | 32 | 1,3 | 1,80E-02 | 0,8 |
| An12g07070 | strong similarity to polyketide synthase PKS1 - Cochliobolus heterostrophus | 38 | 22 | 1,7 | 6,55E-04 | 0,6 |
| An12g07090 | similarity to 6-hydroxy-D-nicotine oxidase 6-HDNO - Arthrobacter oxidans [putative sequencing error] | 19 | 19 | 1,0 | 8,78E-01 | 1,0 |
| An12g07100 | strong similarity to cucumopine synthase cus - Agrobacterium rhizogenes | 20 | 22 | 0,9 | 2,38E-01 | 1,1 |
| An12g07110 | strong similarity to anthranilate synthase component I ybtS - Yersinia pestis | 27 | 28 | 1,0 | 6,63E-01 | 1,0 |
| An12g07120 | similarity to cytochrome P450 monooxygenase P450II - Gibberella fujikuroi | 15 | 15 | 1,0 | 6,48E-01 | 1,1 |
|  |  |  |  |  |  |  |
| An12g07230 | strong similarity to enniatin synthase esyn1 - Fusarium scirpi | 27 | 36 | 0,8 | 3,11E-02 | 1,3 |
|  |  |  |  |  |  |  |
| An13g02380 | weak similarity to hypothetical NADH dehydrogenase subunit 2 ND2 - Lophognathus longirostris | 37 | 37 | 1,0 | 9,70E-01 | 1,0 |
| An13g02390 | similarity to fluconazole resistance protein FLU1 - Candida albicans | 22 | 18 | 1,2 | 5,53E-02 | 0,8 |
| An13g02400 | similarity to nitrate assimilation regulatory protein nirA - Aspergillus nidulans | 312 | 187 | 1,7 | 7,20E-04 | 0,6 |
| An13g02410 | weak similarity to hypothetical cation transporter DRA0361 - Deinococcus radiodurans | 313 | 364 | 0,9 | 2,65E-01 | 1,2 |
| An13g02420 | strong similarity to hypothetical protein EAA54802.1 - Magnaporthe grisea | 28 | 28 | 1,0 | 7,98E-01 | 1,0 |
| An13g02430 | strong similarity to polyketide synthase PKS1 - Cochliobolus heterostrophus | 17 | 17 | 1,0 | 9,15E-01 | 1,0 |
| An13g02450 | strong similarity to hypothetical protein AAO76127.1 - Bacteroides thetaiotaomicron | 33 | 34 | 1,0 | 7,20E-01 | 1,0 |
| An13g02460 | similarity to nonribosomal peptide synthase MxcG - Stigmatella aurantiaca | 98 | 105 | 0,9 | 5,92E-01 | 1,1 |
| An13g02470 | hypothetical protein | 29 | 28 | 1,0 | 8,00E-01 | 1,0 |
| An13g02480 | strong similarity to polyamine oxidase PAO - Zea mays | 169 | 163 | 1,0 | 8,31E-01 | 1,0 |
|  |  |  |  |  |  |  |
| An13g02920 | similarity to polyketide synthase lovF - Aspergillus terreus | 33 | 31 | 1,1 | 5,84E-01 | 0,9 |
| An13g02930 | questionable ORF | 10 | 10 | 1,0 | 7,63E-01 | 1,0 |
| An13g02940 | strong similarity to enoyl reductase of the lovastatin biosynthesis lovC - Aspergillus terreus | 24 | 20 | 1,2 | 8,15E-02 | 0,8 |
| An13g02950 | hypothetical protein | 16 | 15 | 1,1 | 5,35E-01 | 0,9 |
| An13g02960 | strong similarity to lovastatin nonaketide synthase lovB - Aspergillus terreus [truncated ORF] | 21 | 25 | 0,9 | 1,26E-01 | 1,2 |
| An13g02970 | weak similarity to hypothetical protein encoded by An08g03800 - Aspergillus niger | 39 | 40 | 1,0 | 7,36E-01 | 1,0 |
| An13g02980 | similarity to hypothetical protein encoded by An01g08440 - Aspergillus niger | 207 | 247 | 0,8 | 4,79E-01 | 1,2 |
| An13g02990 | similarity to hypothetical protein EAA71271.1 - Gibberella zeae | 35 | 84 | 0,4 | 3,15E-05 | 2,4 |
| An13g03000 | strong similarity to n-alkane-inducible cytochrome P450 protein ALK1 - Yarrowia lipolytica | 91 | 98 | 0,9 | 4,89E-01 | 1,1 |
| An13g03010 | hypothetical protein | 17 | 17 | 1,0 | 9,65E-01 | 1,0 |
| An13g03020 | questionable ORF | 18 | 20 | 0,9 | 1,79E-01 | 1,1 |
| An13g03030 | similarity to hypothetical protein CAB91400.2 - Neurospora crassa [putative sequencing error] | 36 | 41 | 0,9 | 2,41E-01 | 1,1 |
| An13g03040 | strong similarity to enniatin synthase esyn1 - Fusarium scirpi | 22 | 23 | 0,9 | 4,83E-01 | 1,1 |
| An13g03050 | weak similarity to hypothetical protein encoded by An04g04070 - Aspergillus niger | 30 | 47 | 0,6 | 2,14E-02 | 1,6 |
| An13g03060 | strong similarity to ATP-binding cassette multidrug transport protein atrB - Aspergillus nidulans | 105 | 66 | 1,6 | 1,62E-03 | 0,6 |
|  |  |  |  |  |  |  |
| An14g01910 | strong similarity to lovastatin nonaketide synthase lovB - Aspergillus terreus [truncated ORF] | 43 | 44 | 1,0 | 9,44E-01 | 1,0 |
| An14g01920 | questionable ORF | 10 | 10 | 1,0 | 9,58E-01 | 1,0 |
| An14g01930 | questionable ORF | 11 | 11 | 1,0 | 8,46E-01 | 1,0 |
| An14g01940 | strong similarity to enoyl reductase of the lovastatin biosynthesis lovC - Aspergillus terreus | 19 | 20 | 0,9 | 5,99E-01 | 1,1 |
| An14g01950 | similarity to hypothetical protein SPAC18B11.03c - Schizosaccharomyces pombe | 26 | 24 | 1,1 | 5,06E-01 | 0,9 |
| An14g01960 | strong similarity to aberrant X segregation Axs - Drosophila melanogaster | 671 | 840 | 0,8 | 2,31E-02 | 1,3 |
| An14g01970 | similarity to aflatoxin biosynthesis regulatory protein aflR - Aspergillus parasiticus | 27 | 30 | 0,9 | 2,40E-01 | 1,1 |
| An14g01980 | similarity to 2-heptaprenyl-1,4-naphthoquinone methyltransferase menG - Bacillus stearothermophilus | 39 | 46 | 0,8 | 1,38E-01 | 1,2 |
|  |  |  |  |  |  |  |
| An14g04840 | similarity to hypothetical O-methyl transferase EncK - Streptomyces maritimus | 22 | 25 | 0,9 | 3,29E-01 | 1,2 |
| An14g04850 | strong similarity to pimaricin polyketide synthase pimS2 - Streptomyces natalensis | 19 | 17 | 1,1 | 4,23E-01 | 0,9 |
|  |  |  |  |  |  |  |
| An15g04140 | strong similarity to polyketide synthase PKS1 - Cochliobolus heterostrophus | 63 | 814 | 0,1 | 8,15E-07 | 13,0 |
| An15g04150 | strong similarity to oxidoreductase involved in actinorhodin production encoded by Orf11 - Streptomyces lividans | 107 | 1622 | 0,1 | 1,50E-06 | 15,1 |
|  |  |  |  |  |  |  |
| An15g05060 | strong similarity to protein involved in cercosporin production CFP - Cercospora kikuchii | 25 | 25 | 1,0 | 9,99E-01 | 1,0 |
| An15g05070 | similarity to cytochrome P-450 protein Cyp3a-13 - Mus musculus | 26 | 27 | 1,0 | 7,52E-01 | 1,0 |
| An15g05080 | weak similarity to hypothetical protein CAD21084.1 - Neurospora crassa | 87 | 80 | 1,1 | 4,06E-01 | 0,9 |
| An15g05090 | strong similarity to polyketide synthase FUM5 - Gibberella moniliformis | 31 | 31 | 1,0 | 9,58E-01 | 1,0 |
| An15g05100 | similarity to transcription activator Upc2 - Saccharomyces cerevisiae | 43 | 49 | 0,9 | 2,31E-01 | 1,1 |
| An15g05110 | strong similarity to cytochrome P450 monooxygenase P450I - Gibberella fujikuroi | 28 | 30 | 0,9 | 7,48E-01 | 1,1 |
|  |  |  |  |  |  |  |
| An15g07510 | strong similarity to peptide transport gene CaPTR2 - Candida albicans | 18 | 19 | 0,9 | 3,80E-01 | 1,1 |
| An15g07520 | similarity to hypothetical protein mlr2143 - Mesorhizobium loti | 267 | 48 | 5,6 | 4,71E-06 | 0,2 |
| An15g07530 | strong similarity to cyclic peptide AM-toxin synthase AMT - Alternaria alternata | 58 | 58 | 1,0 | 9,97E-01 | 1,0 |
| An15g07540 | hypothetical protein [truncated ORF] | 33 | 35 | 0,9 | 5,96E-01 | 1,1 |
| An15g07550 | strong similarity to neutral amino acid permease mtr - Neurospora crassa | 593 | 751 | 0,8 | 3,77E-02 | 1,3 |
|  |  |  |  |  |  |  |
|  |  |  |  |  |  |  |
| An15g07860 | strong similarity to hypothetical short chain dehydrogenase SPCC736.13 - Schizosaccharomyces pombe | 16 | 18 | 0,9 | 3,34E-01 | 1,1 |
| An15g07870 | strong similarity to alcohol dehydrogenase adhT - Bacillus stearothermophilus | 66 | 67 | 1,0 | 8,64E-01 | 1,0 |
| An15g07880 | strong similarity to hypothetical hydroxylase A - Amycolatopsis orientalis | 24 | 28 | 0,9 | 2,02E-01 | 1,1 |
| An15g07890 | similarity to protein c-fos - Xenopus laevis | 25 | 27 | 0,9 | 5,59E-01 | 1,1 |
| An15g07900 | strong similarity to cytochrome P450 - Myrothecium roridum | 26 | 25 | 1,0 | 9,15E-01 | 1,0 |
| An15g07910 | strong similarity to cyclic peptide AM-toxin synthase AMT - Alternaria alternata | 32 | 32 | 1,0 | 9,27E-01 | 1,0 |
| An15g07920 | strong similarity to lovastatin nonaketide synthase lovB - Aspergillus terreus | 16 | 16 | 1,0 | 9,74E-01 | 1,0 |
| An15g07930 | strong similarity to nitric-oxide synthase - Manduca sexta [truncated ORF] | 17 | 18 | 0,9 | 5,12E-01 | 1,1 |
|  |  |  |  |  |  |  |
| An16g00010 | similarity to alcohol dehydrogenase orfB from patent WO9807867-A2 - Lactococcus lactis | 17 | 20 | 0,8 | 1,88E-01 | 1,2 |
|  |  |  |  |  |  |  |
| An16g00600 | similarity to saframycin Mx1 synthase safA - Myxococcus xanthus | 31 | 26 | 1,2 | 7,99E-02 | 0,8 |
|  |  |  |  |  |  |  |
| An16g01630 | strong similarity to enoyl reductase of the lovastatin biosynthesis lovC - Aspergillus terreus | 237 | 515 | 0,5 | 2,20E-04 | 2,2 |
| An16g01640 | similarity to transcription factor Gal4 - Saccharomyces cerevisiae | 36 | 35 | 1,0 | 9,24E-01 | 1,0 |
| An16g01650 | strong similarity to 1,3,6,8-tetrahydroxynaphthalene reductase arp2 - Aspergillus fumigatus | 33 | 33 | 1,0 | 9,60E-01 | 1,0 |
| An16g01660 | strong similarity to multidrug resistance protein MDR1 - Candida dubliniensis | 21 | 21 | 1,0 | 9,81E-01 | 1,0 |
|  |  |  |  |  |  |  |
| An16g06720 | strong similarity to HC-toxin peptide synthase HTS - Cochliobolus carbonum | 79 | 123 | 0,6 | 1,63E-03 | 1,6 |
|  |  |  |  |  |  |  |
| An16g07170 | similarity to polyketide synthase FUM5 - Gibberella moniliformis [putative frameshift] | 26 | 25 | 1,0 | 8,42E-01 | 1,0 |
| An16g07180 | strong similarity to vanillin dehydrogenase VDH from patent EP0845532 - Unclassified organism | 257 | 178 | 1,4 | 5,08E-03 | 0,7 |
|  |  |  |  |  |  |  |
| An17g00120 | strong similarity to major facilitator superfamily transporter protein mfs1 - Botrytis cinerea | 25 | 28 | 0,9 | 2,50E-01 | 1,1 |
| An17g00130 | weak similarity to cercosporin resistance protein crg1 - Cercospora nictotianae | 28 | 25 | 1,2 | 3,29E-01 | 0,9 |
| An17g00140 | strong similarity to lovastatin nonaketide synthase lovB - Aspergillus terreus [putative frameshift] | 41 | 45 | 0,9 | 4,13E-01 | 1,1 |
|  |  |  |  |  |  |  |
| An18g00480 | strong similarity to cycloheximide resistance protein CYHR - Candida maltosa | 20 | 19 | 1,1 | 5,89E-01 | 0,9 |
| An18g00490 | similarity to salicylate hydroxylase nahW - Pseudomonas stutzeri | 21 | 24 | 0,9 | 1,72E-01 | 1,2 |
| An18g00500 | strong similarity to obtusifoliol 14-alpha demethylase CYP51 - Sorghum bicolor | 19 | 18 | 1,0 | 8,37E-01 | 1,0 |
| An18g00510 | similarity to 6-hydroxy-d-nicotine oxidase 6-HDNO - Arthrobacter oxidans | 34 | 35 | 1,0 | 8,67E-01 | 1,0 |
| An18g00520 | strong similarity to polyketide synthase PKS1 - Cochliobolus heterostrophus | 27 | 31 | 0,9 | 3,52E-01 | 1,1 |
| An18g00530 | strong similarity to versicolorin B synthase vbs - Aspergillus parasiticus | 37 | 38 | 1,0 | 8,82E-01 | 1,0 |
| An18g00540 | similarity to precursor of alpha-latrotoxin - Latrodectus tredecimguttatus | 32 | 26 | 1,2 | 9,42E-02 | 0,8 |
| An18g00550 | strong similarity to O-methyltransferase B omtB - Aspergillus parasiticus | 39 | 40 | 1,0 | 8,20E-01 | 1,0 |
|  |  |  |  |  |  |  |
|  | Fatty acid synthetases Primary metabolism (others are associated with secondary metabolism cluster sabove |  |  |  |  |  |
| An01g00050 | similarity to fatty-acyl-CoA synthase beta chain Fas1 - Saccharomyces cerevisiae [truncated ORF] | 31 | 38 | 0,8 | 2,52E-01 | 1,2 |
| An01g00060 | strong similarity to fatty acid synthase alpha subunit fas2p - Schizosaccharomyces pombe | 1686 | 2195 | 0,8 | 1,68E-02 | 1,3 |
|  |  |  |  |  |  |  |
| An03g00860 | strong similarity to geranylgeranyl pyrophosphate synthase paxG - Penicillium paxilli | 15 | 18 | 0,9 | 2,45E-01 | 1,1 |
|  |  |  |  |  |  |  |
