# Supplementary material for: The Transcriptional Repressor TupA in Aspergillus niger Is Involved in Controlling Gene Expression Related to Cell Wall Biosynthesis, Development, and Nitrogen Source Availability
Source: PLoS One. 2013 Oct 29;8(10):e78102. doi: 10.1371/journal.pone.0078102 (PMC3812127; doi:10.1371/journal.pone.0078102)
Supplement: Table S7 — Expression analysis of PrtT target genes. (DOCX) [file pone.0078102.s009.docx]

**Table S7. Expression of PrtT targets genes in *tupA***

| **Gene ID** | **gene name** | **Description** | ***tupA*** | **WT** | **FC** | **P-value** |
| --- | --- | --- | --- | --- | --- | --- |
| An14g02470 |  | strong similarity to protein PRO304 from patent WO200104311-A1 - Homo sapiens | 117 | 80 | 1,5 | 6,11E-03 |
| An14g04710 | *pepA* | aspartic proteinase aspergillopepsin I pepA - Aspergillus niger | 6375 | 64 | 99,1 | 5,16E-07 |
| An01g00530 | *pepB* | proteinase aspergillopepsin II - Aspergillus niger | 5492 | 27 | 204,0 | 5,15E-08 |
| An06g00190 |  | strong similarity to lysosomal pepstatin insensitive protease CLN2 - Homo sapiens | 305 | 133 | 2,3 | 1,62E-04 |
| An02g04690 |  | strong similarity to serine-type carboxypeptidase I cdpS - Aspergillus saitoi | 2828 | 286 | 9,9 | 5,10E-07 |
| An08g04490 |  | endoprotease Endo-Pro - Aspergillus niger | 114 | 60 | 1,9 | 1,36E-04 |
| An03g05200 |  | strong similarity to carboxypeptidase S1 - Penicillium janthinellum | 1017 | 1022 | 1,0 | 9,74E-01 |
| An08g04640 |  | strong similarity to hypothetical lysosomal pepstatin insensitive protease CLN2 - Canis lupus | 657 | 100 | 6,6 | 1,60E-06 |
| An01g01750 |  | similarity to lysosomal protease CLN2 - Rattus norvegicus | 62 | 51 | 1,2 | 1,74E-01 |
| An04g00410 |  | strong similarity to dipeptidyl peptidase III - Rattus norvegicus | 1144 | 1312 | 0,9 | 2,08E-01 |
| An04g03930 |  | lysine aminopeptidase apsA - Aspergillus niger | 514 | 448 | 1,1 | 1,57E-01 |
| An12g05960 |  | strong similarity to dipeptidyl peptidase II DPPII - Rattus norvegicus | 120 | 128 | 0,9 | 5,39E-01 |
| An16g02560 |  | strong similarity to hypothetical beta-lactamase XF1621 - Xylella fastidiosa | 182 | 135 | 1,3 | 4,10E-02 |

PrtT targets were taken from patent application WO US 2008/0108105 A1 and expression values in the *tupA* mutant and the wild-type strain were retrieved. Several but not not all putative *prtT* targets were higher expressed in the *tupA* strain (higher expressed genes in *tupA* are indicated in yellow)
